# A Gene Regulatory Program for Meiotic Prophase in the Fetal Ovary

**DOI:** 10.1371/journal.pgen.1005531

**Published:** 2015-09-17

**Authors:** Y. Q. Shirleen Soh, Jan Philipp Junker, Mark E. Gill, Jacob L. Mueller, Alexander van Oudenaarden, David C. Page

**Affiliations:** 1 Whitehead Institute, Cambridge, Massachusetts, United States of America; 2 Department of Biology, Massachusetts Institute of Technology, Cambridge, Massachusetts, United States of America; 3 Department of Physics, Massachusetts Institute of Technology, Cambridge, Massachusetts, United States of America; 4 Hubrecht Institute-KNAW (Royal Netherlands Academy of Arts and Sciences) Utrecht, Netherlands; 5 University Medical Center Utrecht, Utrecht, Netherlands; 6 Howard Hughes Medical Institute, Whitehead Institute, Cambridge, Massachusetts, United States of America; Stowers Institute for Medical Research, UNITED STATES

## Abstract

The chromosomal program of meiotic prophase, comprising events such as laying down of meiotic cohesins, synapsis between homologs, and homologous recombination, must be preceded and enabled by the regulated induction of meiotic prophase genes. This gene regulatory program is poorly understood, particularly in organisms with a segregated germline. We characterized the gene regulatory program of meiotic prophase as it occurs in the mouse fetal ovary. By profiling gene expression in the mouse fetal ovary in mutants with whole tissue and single-cell techniques, we identified 104 genes expressed specifically in pre-meiotic to pachytene germ cells. We characterized the regulation of these genes by 1) retinoic acid (RA), which induces meiosis, 2) *Dazl*, which is required for germ cell competence to respond to RA, and 3) *Stra8*, a downstream target of RA required for the chromosomal program of meiotic prophase. Initial induction of practically all identified meiotic prophase genes requires *Dazl*. In the presence of *Dazl*, RA induces at least two pathways: one *Stra8*-independent, and one *Stra8*-dependent. Genes vary in their induction by *Stra8*, spanning fully *Stra8*-independent, partially *Stra8*-independent, and fully *Stra8*-dependent. Thus, *Stra8* regulates the entirety of the chromosomal program but plays a more nuanced role in governing the gene expression program. We propose that *Stra8*-independent gene expression enables the stockpiling of selected meiotic structural proteins prior to the commencement of the chromosomal program. Unexpectedly, we discovered that *Stra8* is required for prompt down-regulation of itself and *Rec8*. Germ cells that have expressed and down-regulated *Stra8* are refractory to further *Stra8* expression. Negative feedback of *Stra8*, and subsequent resistance to further *Stra8* expression, may ensure a single, restricted pulse of *Stra8* expression. Collectively, our findings reveal a gene regulatory logic by which germ cells prepare for the chromosomal program of meiotic prophase, and ensure that it is induced only once.

## Author Summary

The formation of haploid gametes from diploid germ cells requires a specialized reductive cell division known as meiosis. This reductive division is enabled by chromosomal events that occur during meiotic prophase, including synapsis and crossing-over of homologous chromosomes. These chromosomal events involve meiosis-specific genes that must be expressed before they act during meiosis. Using gene expression profiling, we identified a set of mammalian meiosis-specific genes. To understand how expression of these genes is controlled, we examined their expression in the absence of known regulators of the chromosomal events: 1) retinoic acid (RA), which induces meiosis, 2) *Dazl*, which is required for germ cell competence to respond to RA, and 3) *Stra8*, which is induced by RA and is required for the chromosomal events of meiotic prophase. We uncover two key features of gene regulation. First, while the genes require RA and *Dazl* to be expressed, they vary in their dependence on *Stra8*, thus creating a regulatory hierarchy. Genes induced independently of *Stra8*, and thus early in this hierarchy, may encode proteins that are stockpiled in anticipation of the chromosomal events. Second, *Stra8* induces its own down-regulation, and may thus prevent repeated induction of meiosis in a single germ cell.

## Introduction

In sexually reproducing organisms, germ cells undergo meiosis, a specialized cell division program that produces haploid gametes. The reductive segregation of chromosomes depends upon a complex series of chromosomal events that occur during meiotic prophase. This chromosomal program must be supported by expression of a large suite of genes. A genome-wide description of this gene expression program, and how it is regulated, has not been available for mammals or other animals with specialized sex cells, or germ cells. Indeed, the best existing model for such a gene regulatory program is that of budding yeast [1–4].

The chromosomal program of meiotic prophase, including events such as laying down of meiotic cohesins, synapsis between homologs, and homologous recombination, has been the subject of intense study [5–7]. Investigations of these processes in mammals have relied principally upon identifying mouse orthologs of proteins that have demonstrated meiotic functions in lower eukaryotes, and that are well conserved among sexually reproducing species [8,9]. However, not all proteins involved in the meiotic chromosomal processes are well conserved among eukaryotes, and identifying these exceptions has proven challenging [10]. Identification of a gene set specific to mammalian meiotic prophase would provide an orthogonal means of discovering poorly conserved or even novel proteins involved in the chromosomal program of mammalian meiotic prophase.

Studies in a mammalian system are also required if we are to understand how the gene expression program of mammalian meiotic prophase is regulated; the regulation of meiotic initiation is poorly conserved. For instance, between mouse and budding yeast, the regulatory logic of meiotic initiation appears similar, but the molecular identities of the regulators are not conserved [11]. In both mouse ovarian and testicular germ cells, meiosis is initiated by retinoic acid (RA) [12–14], a signaling molecule restricted to chordates [15]. RA induces *Stra8*, a vertebrate-specific gene that encodes a putative helix-loop-helix-containing transcription factor [13,14,16–20]. *Stra8* is required for all chromosomal events of meiotic prophase assayed, including cohesion, synapsis, and recombination, as well as the preceding meiotic DNA replication [12,20]. In mouse fetal ovarian germ cells, induction of *Stra8* by RA requires the germ-cell-expressed competence factor *Dazl* [21]. *Dazl*, which encodes an RNA binding protein expressed in postmigratory XX and XY germ cells, is required for germ cells to gain competence to respond to developmental cues, including RA [22]. Thus far, the roles of RA, *Stra8*, and *Dazl* have largely been assayed with respect to the chromosomal program of meiotic prophase; their potential roles in regulating the gene expression program have not been examined systematically.

We sought to elucidate the gene regulatory program of meiotic prophase. We used the mouse fetal ovary as a model for two reasons. First, germ cells in the fetal ovary initiate and progress through meiotic prophase with greater synchrony than in the postnatal or adult testis. All germ cells in the fetal ovary initiate meiosis around embryonic day 13.5, progress through meiotic prophase during subsequent fetal development, and arrest at diplotene of meiotic prophase before birth. Initiation and progression of meiotic prophase occurs in an anterior-to-posterior wave: expression of *Stra8* and meiotic prophase genes begins in the anterior portion of the fetal ovary before extending towards the posterior [23,24]. We therefore took advantage of the relative synchrony of cell state over time and space to finely dissect initiation and progression of meiotic prophase. Second, the roles of *Dazl*, RA, and *Stra8* in meiotic initiation are well defined in the fetal ovary. To determine how *Dazl*, RA, and *Stra8* regulate the gene expression program, we profiled expression in wild-type and mutant animals. We used whole-gonad, genome-wide transcriptome profiling, to obtain a global description of gene expression, and followed up with targeted single-cell, single-transcript measurements to precisely quantify elements of regulatory control at the level of individual germ cells.

We identified a set of 104 genes specific to meiotic prophase, as assayed in fetal ovarian germ cells. We characterized how *Dazl*, RA, and *Stra8* regulate this gene expression program, thus complementing our previous understanding of how they regulate the chromosomal program. From these data, we discerned two elements of gene regulatory logic centered on *Stra8*, a key inducer of the chromosomal program. Initial induction of genes requires *Stra8*-independent and *Stra8*-dependent pathways. After gene induction, *Stra8* is required for subsequent down-regulation of its own expression. We propose that these elements of gene regulatory logic account for how germ cells prepare for and ensure a single induction of the chromosomal program of meiotic prophase.

## Results

### Identification of 104 genes specific to meiotic prophase

**Fig 1:**
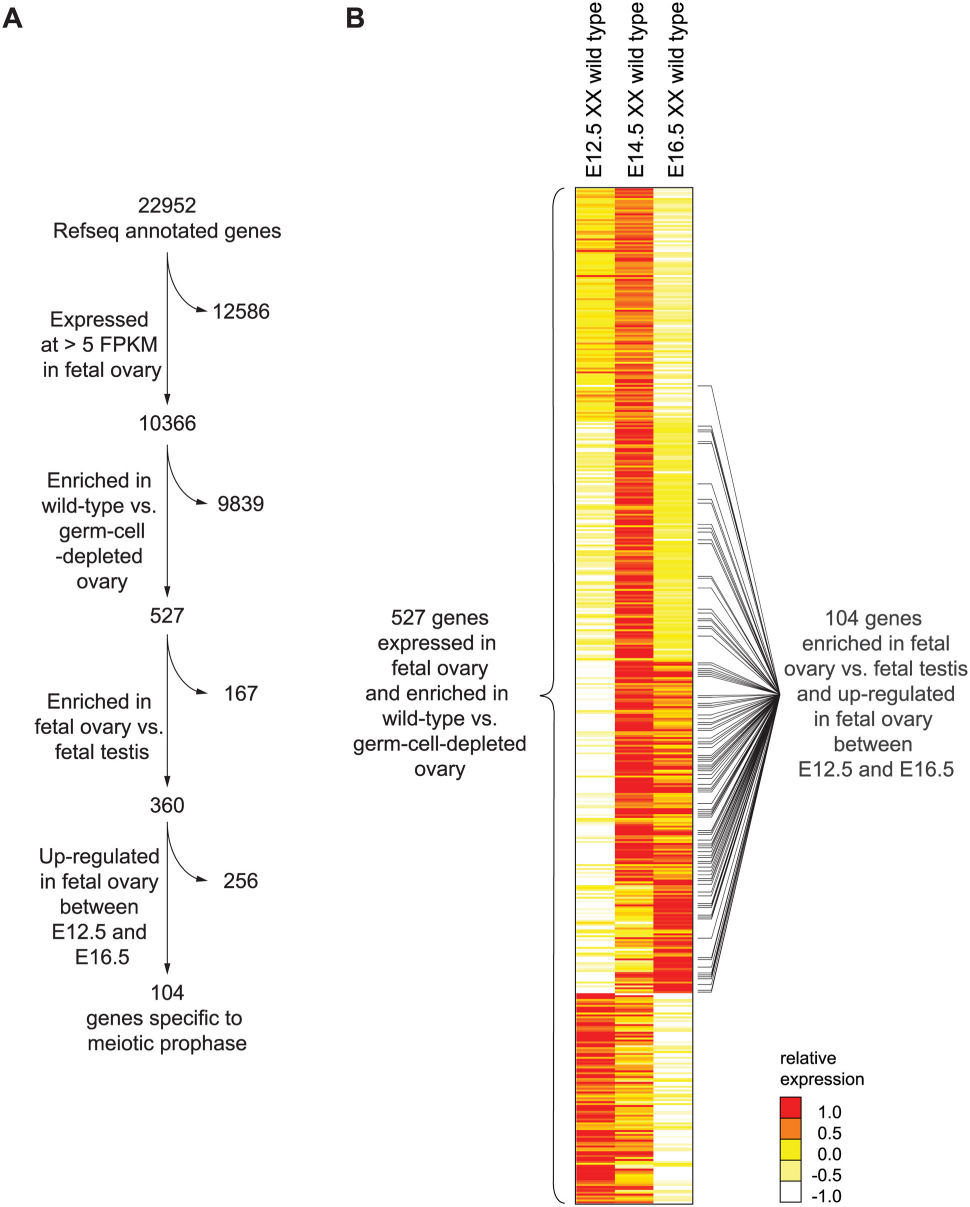
RNA-seq analysis of wild-type and germ-cell-depleted fetal gonads identifies a meiotic prophase-specific gene set. (A) Four filters were used to identify a set of genes expressed specifically in meiotic prophase. First, genes were required to be expressed in wild-type fetal ovary (E12.5, E14.5, or E16.5) at >5 FPKM; a total of 10,366 genes met this criterion. Of these 10,366 genes, 526 were also expressed more highly in wild-type than in germ-cell-depleted fetal ovary (*Kit* ^*W*^/*Kit* ^*Wv*^) (fold-change > 2, FDR adjusted p value, q <0.01 at either E12.5, E14.5, or E16.5). As explained in the text, *Rec8* was also included, for a total of 527 genes. Of these 527 genes, 360 were expressed more highly in fetal ovary than in fetal testis (fold-change > 2, q < 0.01). Of these 360 genes, 104 were up-regulated between E12.5 and E14.5 or E16.5 in the ovary (fold-change > 2, q < 0.01). (B) Relative expression of 527 ovarian germ-cell-enriched genes in E12.5, E14.5, and E16.5 wild-type (*Kit* ^+^/*Kit* ^+^) ovary. Gene expression is represented as log transformed and mean centered FPKM. Genes (rows) are organized by k-means clustering (k = 5). Black bars to right of gene-expression heat map represent the 104 genes that were both up-regulated between E12.5 and E14.5 or E16.5 in the ovary, and expressed more highly in fetal ovary than in fetal testis. Source data for the 527 genes, as well as all other Refseq genes, are provided in S1 Table.

To identify and catalog the gene expression program of meiotic prophase as it occurs in the fetal ovary, we performed genome-wide transcriptome profiling by RNA-seq on whole fetal ovaries at embryonic days 12.5, 14.5, and 16.5 (E12.5, E14.5, and E16.5). At these time points, ovarian germ cells are in pre-meiotic, leptotene (early meiotic prophase), and pachytene (mid-late meiotic prophase) stages, respectively [25,26]. At each time point, we assayed expression in gonads of wild-type and germ-cell-depleted (*Kit* ^*W*^/*Kit* ^*Wv*^) mice [27], in order to identify germ-cell-dependent genes. During this embryonic period, testicular germ cells do not initiate meiosis, but instead enter and remain in mitotic G0/G1 arrest [28]. We therefore also profiled expression from wild-type fetal testes, at the same time points, to identify ovary-enriched genes. We defined “meiotic prophase genes” as those meeting the criteria summarized in Fig 1A.

Since genes involved in meiotic prophase should be expressed leading up to or during prophase, we required that genes be expressed in wild-type ovaries at one or more of the three time points (E12.5, E14.5, or E16.5) at greater than 5 Fragments Per Kilobase of transcript per Million mapped reads (FPKM). We found 10,366 genes to be expressed at this level in the fetal ovary (Fig 1A). Additionally, since only germ cells might be expected to express genes required for meiotic prophase, we also required that gene expression be at least 2-fold higher in wild-type than in germ-cell-depleted ovaries. A total of 526 genes met both criteria (Fig 1A, S1 Table). There was one conspicuous absence: *Rec8*, a meiotic cohesin, was not germ-cell-enriched. We verified by single-molecule fluorescent in situ hybridization that *Rec8* was indeed expressed in ovarian somatic cells as well as germ cells (S1 Fig), and added it to the 526 genes.

Using k-means clustering, we identified several distinct gene expression profiles (Fig 1B). Of the 527 genes, about 46%, including *Stra8*, were up-regulated between E12.5 and E14.5, then down-regulated by E16.5, suggestive of functions restricted to early meiotic prophase. About 22% of the 527 genes were up-regulated between E12.5 and E14.5 and remained elevated at E16.5; these genes include *Sycp3*, which encodes a synaptonemal complex protein. About 11% of genes were not up-regulated until E16.5, suggestive of functions later in meiotic prophase. The remaining 21% of the 527 genes were highly expressed at E12.5 and progressively down-regulated by E16.5. These include many pluripotency markers, including *Pou5f1* (*Oct4*), *Nanog*, and *Sox2*, and reflect the down-regulation of a pluripotency program as germ cells enter meiosis [29–31].

To winnow this list of 527 ovarian germ cell genes down to those functioning in meiotic prophase, we required two additional criteria (Fig 1A). Since testicular germ cells do not embark on meiosis until well after birth, we required that gene expression be at least 2-fold higher in fetal ovary than in fetal testis; 360 genes met this additional criterion. Since genes with meiotic functions should be up-regulated as germ cells enter and progress through meiosis, we also required that genes be at least 2-fold up-regulated between E12.5 and E14.5 or E16.5; 104 genes satisfied this as well as the earlier criteria. We refer to this final set of 104 genes as the gene expression program of meiotic prophase.

Of these 104 genes, 54 have previously been implicated in meiotic prophase by independent, lower-throughput methods. For 33 of these 104 genes, loss-of-function mutants have been examined for fertility defects; defects in meiotic prophase or fertility were reported for 32 of the 33 genes tested in this manner (S1 Text). For 21 of the remaining 71 genes, detailed descriptions of RNA or protein expression patterns are publicly available, and all are consistent with functions in meiotic prophase. Thus, among 104 genes implicated in meiotic prophase through our systematic whole-genome RNA-seq analysis, 53 (of 54 genes tested) are substantiated by prior studies.

These findings suggest that many of the remaining 50 (of 104) genes are novel and uncharacterized genes involved in meiotic prophase, representing a great opportunity for future study. Review of the published literature indicates that our RNA-seq analysis captured most meiotic prophase genes that are expressed specifically in meiotic germ cells. Of 21 genes for which mutant germ cells have been reported to arrest at leptotene, zygotene, or pachytene stages of meiotic prophase (as cataloged by Handel and Schimenti, 2010), 14 are represented in our list of 104 genes. The seven genes with meiotic prophase arrest phenotypes that we failed to identify by RNA-seq analysis are either ubiquitously expressed (such as *Cyclin-dependent kinase 2*, *Cdk2*) or are expressed in both ovarian and testicular germ cells (such as *Piwi-like RNA-mediated gene silencing 2*, *Piwil2*). The design of our study would preclude our identifying genes that are expressed in both ovarian germ cells and somatic cells, or that are expressed at substantial levels in fetal testes.

### Meiotic prophase gene expression is wholly dependent on *Dazl*, but ranges from completely *Stra8*-dependent to *Stra8*-independent

**Fig 2:**
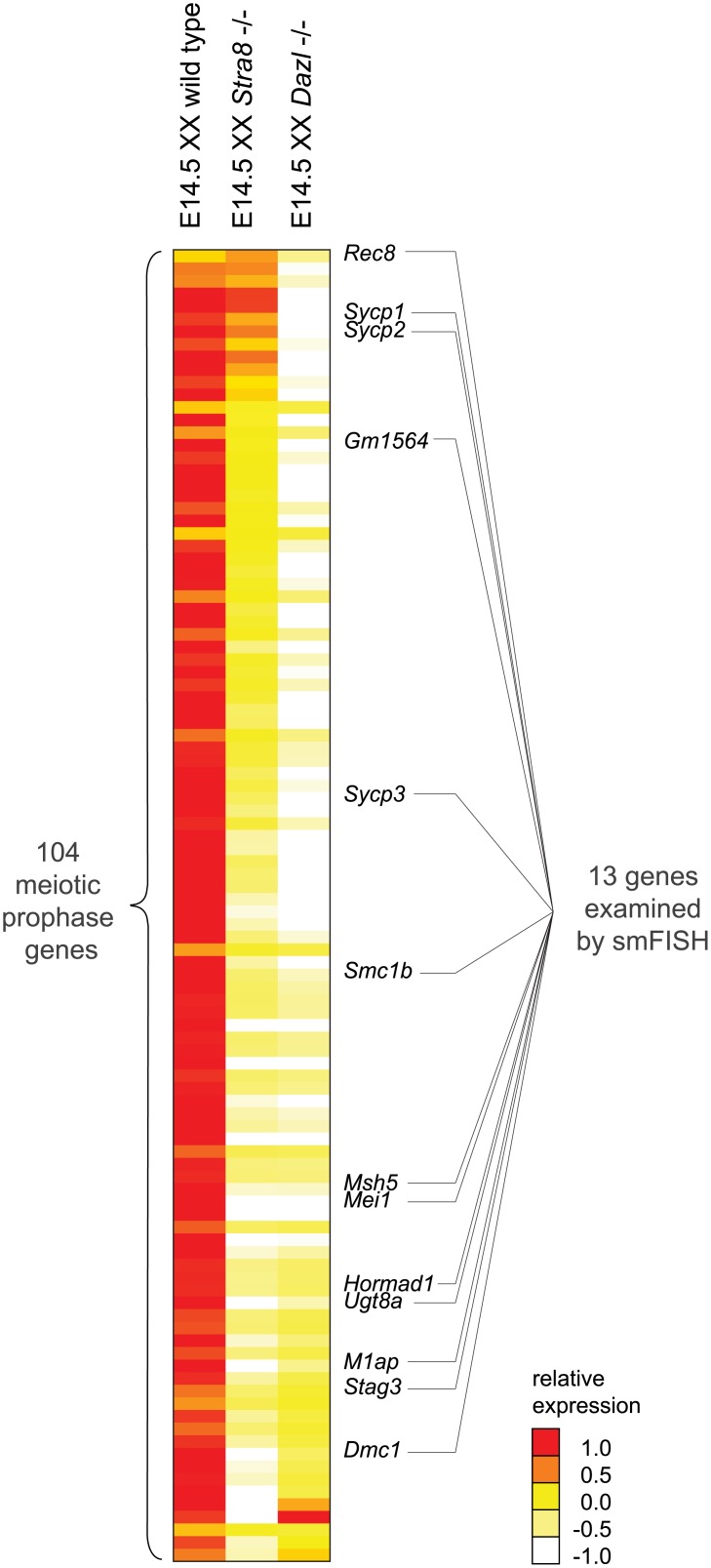
RNA-seq analysis of *Dazl* and *Stra8*-deficient fetal gonads reveals *Stra8*-independent regulation of meiotic prophase genes. Relative expression of 104 meiotic prophase-specific genes in E14.5 wild-type, *Stra8*-deficient, and *Dazl*-deficient ovary. Gene expression was measured by RNA-seq and represented as log transformed and mean centered FPKM. Genes (rows) are arranged from least to most down-regulated in the *Stra8*-deficient ovary relative to the *Dazl*-deficient ovary, with the exception of the bottom four genes which were not significantly down-regulated in the *Dazl*-deficient ovary (q > 0.05), and were expressed at < 5 FPKM in wild-type, *Dazl*-deficient, and *Stra8*-deficient ovaries. Thirteen genes, listed to the right of the gene expression heat map, were selected for subsequent smFISH analysis. Source data for the 104 meiotic prophase genes, as well as all Refseq annotated genes, is provided in S2 Table.

We next sought to determine how the meiotic prophase genes are activated. *Stra8* was previously shown to be required for meiotic initiation, as primarily assayed by the meiotic chromosomal program. *Dazl* and RA are germ-cell-intrinsic and -extrinsic factors, respectively, required for induction of *Stra8* and initiation of the chromosomal program. The roles of these factors in regulating the program of gene expression are largely unknown. We first determined whether *Dazl* and *Stra8* regulate meiotic prophase genes by examining gene expression by RNA-seq in whole E14.5 *Dazl*-deficient (*Dazl*-/-) and *Stra8*-deficient (*Stra8* -/-) ovaries as compared to corresponding homozygous wild-type controls (Fig 2, S2 Table).

*Dazl* is required for germ cells to acquire competence to respond to RA. *Dazl*-expressing germ cells respond to RA by expressing *Stra8* and initiating meiosis. *Dazl* is also more broadly required for the processes of gametogenesis, which encompass meiosis, the sex-specific cellular differentiation events of oogenesis and spermatogenesis, and the down-regulation of pluripotency markers [21,22]. Given *Dazl*’s broad role in competence for gametogenesis, we predicted that *Dazl* would be required for induction of the meiotic prophase gene expression program. Indeed, we found that expression of practically all meiotic prophase genes (100 of 104) was significantly diminished if not eliminated in *Dazl*-deficient ovaries (Fig 2, S2 Table). The remaining four genes were expressed at < 5 FPKM in both wild-type and *Dazl*-deficient ovaries.

*Stra8* is required for the chromosomal program of meiotic prophase, including loading of meiotic cohesins, such as REC8, and assembly of the synaptonemal complex proteins, including SYCP3. However, although the REC8 and SYCP3 proteins do not localize to chromosomal axes in *Stra8*-deficient germ cells, the proteins are nevertheless produced [20]. In fact, *Rec8* expression can be induced in testicular germ cells by RA in the absence of *Stra8* function [32]. These results suggest that while *Stra8* might regulate the entirety of the chromosomal program, it might have a more limited role in governing the gene expression program. We aimed to clarify the extent to which *Stra8* regulates the meiotic gene expression program.

We found that expression of the 100 *Dazl*-dependent genes ranged across a wide spectrum of *Stra8*-dependency. For slightly over half of the 100 genes, including *Dmc1*, which is required to repair meiotic double-strand breaks, expression appeared to be fully dependent on *Stra8*. Expression of these genes was reduced in *Stra8*-deficient ovaries to levels as low as in the *Dazl*-deficient ovary (Fig 2, S2 Table). Expression of the remaining genes appeared to be partially dependent on, or in a few cases, largely independent of *Stra8*. Some genes, such as *Sycp3*, were expressed at lower levels in *Stra8*-deficient ovaries than in wild-type ovaries, but still at higher levels than in *Dazl*-deficient ovaries. At the *Stra8*-independent extreme of the spectrum is *Rec8*, whose levels were not only undiminished in *Stra8*-deficient ovaries, but in fact were modestly increased.

Thus, RNA-seq analyses of whole *Dazl*-deficient and *Stra8*-deficient ovaries suggest a model of gene induction whereby *Dazl* is required for induction of the meiotic prophase gene expression program via at least two pathways: a *Stra8*-independent pathway, and a *Stra8*-dependent pathway.

### *Stra8*-independent and *Stra8*-dependent pathways act additively in individual cells

Whole-gonad RNA-seq analysis provides genome-wide breadth in characterizing the gene expression program of meiotic prophase. However, because this method averages across a population that includes a diversity of both germ cells and somatic cells, our observations may not accurately reflect events in individual germ cells. Specifically, we wondered whether our observation that some genes appeared partially *Stra8*-independent by RNA-seq actually reflected a partial reduction in gene expression in all *Stra8*-deficient cells. If so, this would indicate that *Stra8*-dependent and *Stra8*-independent pathways act additively in individual germ cells. Alternatively, our RNA-seq observation could be explained by a subset of *Stra8*-deficient germ cells retaining wild-type levels of gene expression, with other *Stra8*-deficient germ cells having greatly reduced levels of gene expression.

**Fig 3:**
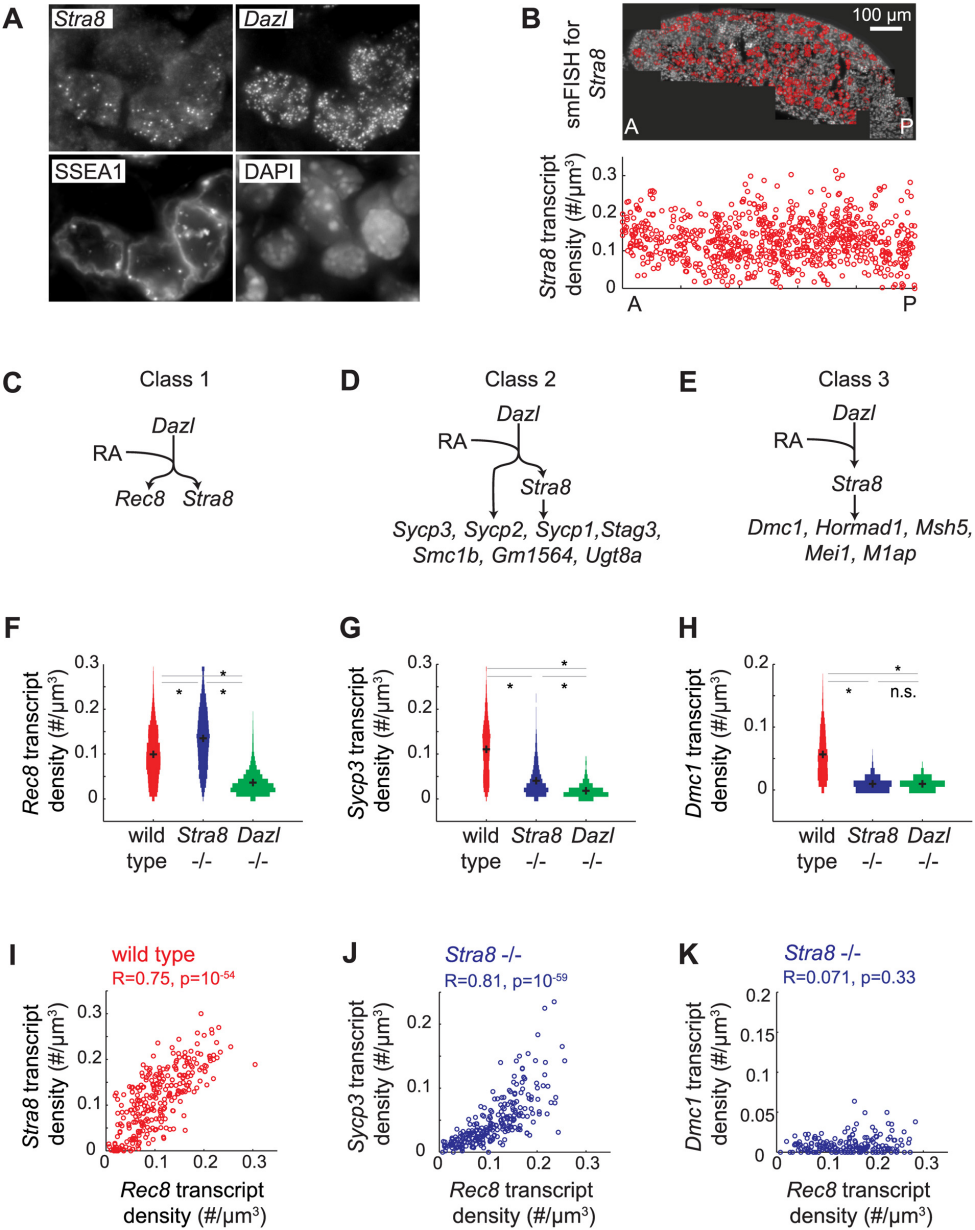
Single molecule FISH analysis corroborates three classes of gene regulation at the level of individual germ cells. (A) Detection of single transcripts of *Stra8* and *Dazl* by smFISH in E14.5 fetal ovary. Single transcripts are visible as punctate signals. Germ cells are co-stained for SSEA1, and DAPI. (B) Top: Individual images are stitched together for an entire E14.5 ovary. Red signal in the stitched image represents *Stra8* transcript. Bottom: Scatterplot of *Stra8* transcript density in single germ cells, along the anterior-posterior axis of the ovary. Transcript density is the number of transcripts per cell normalized by cell size (#/um^-3^). (C), (D), (E) Models for three classes of regulation, and classification of 13 representative meiosis genes into the three classes by smFISH data. (C) Genes that are fully *Stra8*-independent, (D) genes that require both a *Stra8*-independent and a *Stra8*-dependent pathway to be fully expressed, and (E) genes that are fully *Stra8*-dependent. (F), (G), (H) Distributions of transcript densities in E14.5 wild-type (red), *Stra8*-deficient (blue) and *Dazl*-deficient (green) ovarian germ cells, for (F) *Rec8*, a representative of Class 1, (G) *Sycp3*, a representative of Class 2, and (H) *Dmc1*, a representative of Class 3. Asterisks represent significant differences between the means of the distributions (p< 0.05, t-test on average transcript densities of biological replicates). (I) Representative scatterplot of transcript densities of *Stra8* against *Rec8* in E14.5 wild-type ovarian germ cells. (J), (K) Representative scatterplots of transcript densities of *Sycp3* against *Rec8* (J), and *Dmc1* against *Rec8* (K) in E14.5 *Stra8*-deficient ovarian germ cells. (I), (J), (K) Correlation coefficients for biological replicates are provided in S3 Table.

Distinguishing between these two scenarios required measurement of gene expression with single-cell resolution. We used single molecule fluorescence in situ hybridization (smFISH) to quantify gene expression in single cells in situ. smFISH involves multiple short fluorescently-labeled oligonucleotide probes that collectively bind along the same target transcript to detect and localize each target mRNA molecule as a punctate signal [33] (Fig 3A). These punctate signals can be quantified to determine the number of transcripts per cell volume (transcript density) (Fig 3B). We selected 13 genes, spanning a spectrum of *Stra8*-dependencies as measured by RNAseq in E14.5 ovaries (Fig 2), for examination by smFISH in germ cells of E14.5 wild-type, *Stra8*-deficient, and *Dazl*-deficient ovaries. Selected genes include the meiotic-specific cohesins *Rec8*, *Smc1b*, and *Stag3*; the synaptonemal complex proteins *Sycp1*, *Sycp2*, and *Sycp3*; *Dmc1* and *Msh5*, which are involved in double-strand break repair; and *Hormad1*, which promotes homolog alignment and synaptonemal complex formation. We also included *Mei1* and *M1ap*, which exhibit defects in meiotic prophase when mutated, and *Gm1564* and *Ugt8a*, which are presently uncharacterized.

Our smFISH studies of individual germ cells confirmed that all 13 genes were *Dazl*-dependent, and that they ranged across a spectrum of *Stra8*-dependence. For conceptual simplification and ease of discussion, we will describe this spectrum as comprising three classes: Class 1 genes—fully *Stra8*-independent, Class 2 genes—partially *Stra8*-independent, and Class 3 genes—fully *Stra8*-dependent (Fig 3C–3E). *Rec8* fell into Class 1, fully independent of *Stra8* expression (Fig 3C and 3F). Expression of *Rec8* in *Stra8*-deficient germ cells, as a population, was in fact slightly higher than in wild type, an observation we later explored. *Dmc1*, *Msh5*, *Hormad1*, *Mei1*, and *M1ap* fell into Class 3, fully dependent on *Stra8* (Fig 3E and 3H, S2A Fig). Their expression in *Stra8*-deficient germ cells was reduced (compared to wild-type germ cells) to the same degree as in *Dazl*-deficient germ cells. *Sycp3*, *Sycp2*, *Sycp1*, *Stag3*, *Smc1b*, *Gm1564*, and *Ugt8a* fell into Class 2, partially independent of *Stra8* expression (Fig 3D and 3G, S2A Fig). Their expression in *Stra8*-deficient germ cells was, as a population, significantly lower than in wild type, but significantly higher than in *Dazl*-deficient germ cells. We always observed a unimodal distribution of gene expression, which is consistent with gene expression being reduced in each germ cell. The direction and relative magnitude of gene expression changes as measured by smFISH and RNA-seq are consistent for all 13 genes (S2B Fig).

### Single-cell correlation of *Stra8*-independent gene expression with expression of RA-induced genes

What is the role of RA in regulating the meiotic prophase genes? It was previously shown that RA induces *Stra8* expression in fetal ovarian germ cells [13,14]. Therefore, *Stra8*-dependent induction of Class 2 and 3 genes would depend, indirectly, on RA. Does RA also regulate the *Stra8*-independent induction of Class 1 and 2 genes? We previously showed that RA induces *Rec8* in the absence of *Stra8* [32], and we now demonstrate, quantitatively, the full independence of *Rec8* expression from *Stra8*. By extension, we hypothesized that RA is responsible for *Stra8*-independent induction of not just *Rec8*, a Class 1 gene, but also of the Class 2 genes.

An ideal test of this hypothesis would be to eliminate RA *in vivo* in the fetal ovary. This was not technically feasible, so we instead sought evidence of RA regulation by analyzing gene expression in hundreds of individual germ cells, and using endogenous variation in expression of an RA-induced gene in these hundreds of germ cells as a read out of cell response to RA. If variation in expression of an RA-induced gene reflects the individual cell’s response to RA, then expression of two RA-induced genes across hundreds of individual germ cells should be positively correlated. To test this, we examined variation in expression of the two known independently RA-induced genes, *Stra8* and *Rec8*. We found that *Rec8* transcript density is indeed positively correlated with *Stra8* transcript density in germ cells of E14.5 fetal ovaries (Fig 3I).

We then employed variation in the level of *Rec8* expression as a quantifiable read out of RA response, so as to determine if *Stra8*-independent expression of genes is due to RA. If so, then expression of the gene, in the absence of *Stra8*, should be correlated with that of *Rec8*. We quantified expression of each Class 2 gene alongside *Rec8*, in hundreds of individual *Stra8*-deficient germ cells at E14.5. Expression of *Sycp3*, *Sycp2*, *Sycp1*, *Stag3*, *Gm1564*, and *Ugt8a* is positively correlated with *Rec8* expression (Fig 3J, S3 Fig, S3 Table). As expected, for Class 3 genes, which are fully *Stra8*-dependent, residual expression in the absence of *Stra8* did not correlate with *Rec8* expression (Fig 3K, S3 Fig, S3 Table). These results are consistent with the *Stra8*-independent pathway being regulated by RA, either directly or indirectly.

### *Stra8*-independent pathway enables maximal and early gene expression

Our model of gene regulation inferred from E14.5 fetal ovaries led us to predict two consequences for gene expression over time. First, we reasoned that, for Class 2 genes, both *Stra8*-independent and *Stra8*-dependent pathways might be required to attain maximal levels of gene expression. If so, expression of Class 2 genes in *Stra8*-deficient germ cells would not reach peak wild-type levels, even after an extended period of time. Second, we considered the possibility that RA-dependent, *Stra8*-independent induction of Class 1 and 2 genes might function to induce genes that are required early in meiotic prophase, in anticipation of *Stra8*. If so, Class 1 and 2 genes might be induced in parallel with *Stra8*, and before Class 3 genes.

To determine the temporal dynamics of gene expression with fine resolution, we took advantage of previous observations that fetal ovarian germ cells initiate and progress through meiotic prophase in an anterior-to-posterior wave [23,24]. *Stra8*, *Dmc1*, and *Sycp3* expression have been observed to be induced first in germ cells in the anterior portion of the fetal ovary, and only later in the posterior. Therefore, measuring gene expression as a function of anterior-posterior position in addition to time should provide finer resolution of events than would time alone. To formally test the hypothesis that the anterior-posterior axis is a proxy for time, we compared expression changes of 527 germ-cell-enriched genes over time (between E12.5 and E13.5, anterior third of ovaries only), and over space (between posterior and anterior thirds of E13.5 ovaries). We found that gene expression changes over both time and space were indeed highly correlated (S4 Fig, S4 Table), validating our spatiotemporal approach.

**Fig 4:**
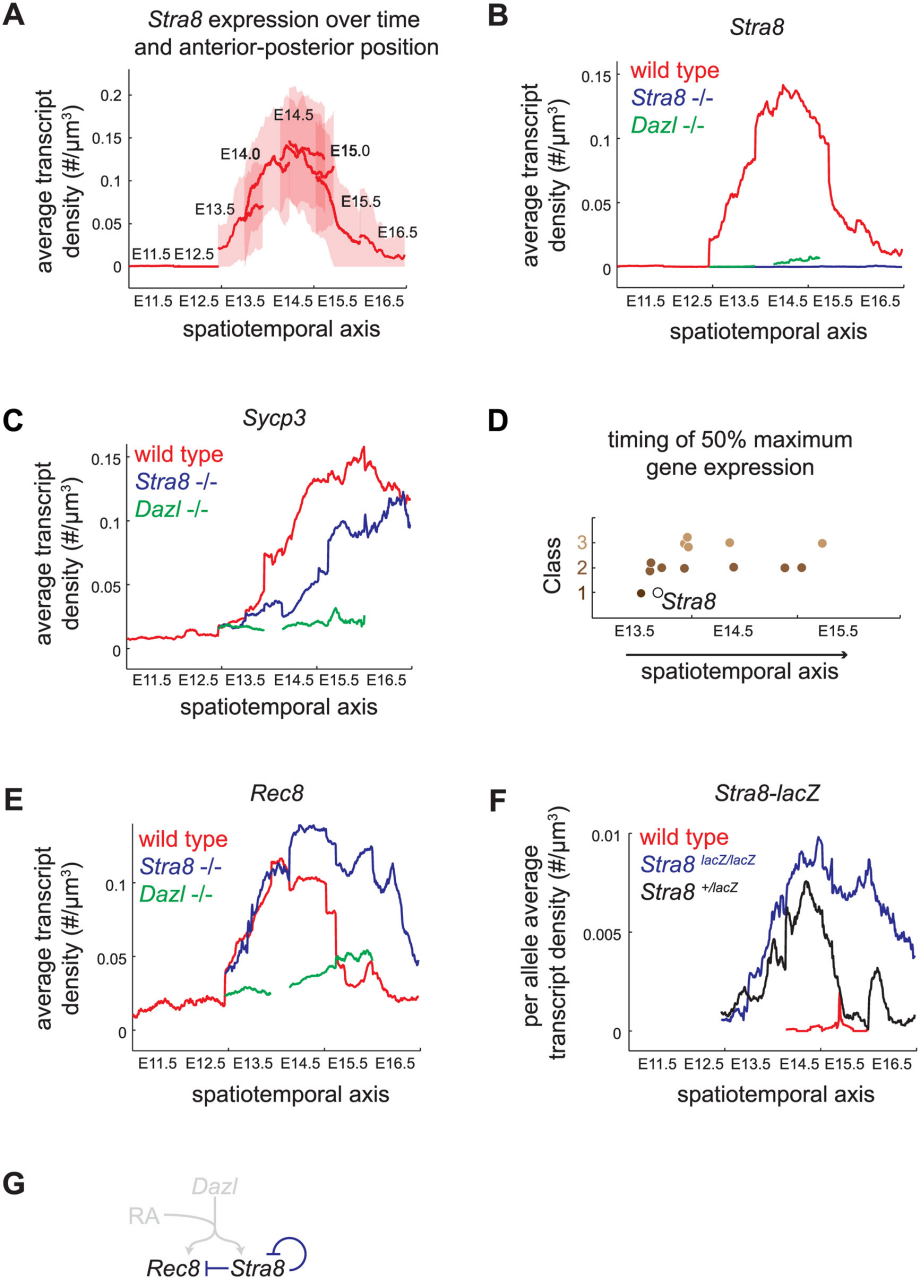
Spatiotemporal analysis demonstrates role of *Stra8*-independent pathway in inducing maximal and early gene expression, and identifies *Stra8*-dependent down-regulation of *Stra8* and *Rec8*. (A) Construction of spatiotemporal plot of *Stra8* average transcript densities along the anterior-posterior axis of ovary at E11.5, E12.5, E13.5, E14.0, E14.5, E15.0, E15.5, and E16.5. To construct a smooth average transcript density trace, time points are overlapped based on *Stra8* transcript density levels. Bold line indicates mean of distribution, light band indicates 1 standard deviation from the mean. (B) Spatiotemporal plot of *Stra8* expression in wild-type (red), *Stra8*-deficient (blue) and *Dazl*-deficient (green) germ cells. (C) Spatiotemporal plot of *Sycp3* expression in wild-type (red), *Stra8*-deficient (blue) and *Dazl*-deficient (green) germ cells. (D) Timing of 50% maximal induction of genes for Class 1, 2, and 3 genes (filled brown circles). Time is represented by a spatiotemporal axis (x-axis), same as in (A). Time of 50% *Stra8* induction is represented by open black circle. (E) Spatiotemporal plot of *Rec8* expression in wild-type (red), *Stra8*-deficient (blue) and *Dazl*-deficient (green) germ cells. *Rec8* average transcript densities are significantly higher in *Stra8*-deficient compared to wild-type germ cells at E14.5, E15.5, and E16.5 (p< 0.05, t-test on average transcript densities of biological replicates). (F) Spatiotemporal plot of *lacZ* expression from the endogenous *Stra8* locus in *Stra8*-deficient (blue) and *Stra8* heterozygote (wild type/*lacZ*) (black) germ cells, normalized per allele. Normalized *lacZ* transcript densities differ significantly at E15.5, and E16.5 (p< 0.05, t-test on average transcript densities of biological replicates). (G) Model representing *Stra8*-dependent down-regulation of *Stra8* and *Rec8*.

We assayed gene expression over a spatiotemporal axis, using *Stra8* expression in wild-type germ cells as a reference, as follows. We measured the transcript density of *Stra8* in individual germ cells at E11.5, E12.5, E13.5, E14.0, E14.5, E15.0, E15.5, and E16.5. For each time point, we calculated the average transcript density along the longitudinal axis, from the posterior pole (germ cells at least advanced state) to the anterior pole (germ cells at most advanced state) (Fig 4A). We then joined these average expression traces from consecutive time points to create a continuous trace of average transcript density along a spatiotemporal axis (Fig 4B). Using this approach, we quantified the *Stra8* pulse of expression in the wild-type ovary, which was previously observed semi-quantitatively, by whole-mount in situ hybridization [23].

We applied this spatiotemporal analysis to characterize expression dynamics of the subset of 13 meiotic prophase genes in wild-type, *Stra8*-deficient, and *Dazl*-deficient germ cells. First, we asked if Class 2 genes indeed required both *Stra8*-independent and *Stra8*-dependent pathways to attain maximal levels of gene expression. We found that, in *Stra8*-deficient germ cells, Class 2 genes failed to reach expression levels seen in wild type even when given an additional one to two days after expression peaks in wild type. For example, expression of *Sycp3* in *Stra8*-deficient germ cells had begun to decline by E16.5, without having reached the peak levels of expression achieved (at E15.5) in wild-type germ cells (Fig 4B, S5 Fig). Thus, the *Stra8*-independent pathway is crucial to ensure full expression of Class 2 genes.

Second, we asked if *Stra8*-independent induction of Class 1 and 2 genes might enable early gene expression. We found that induction of four Class 1 and 2 genes–*Rec8*, *Stag3*, *Smc1b*, and *Gm1564* –indeed occurred early, and contemporaneous with *Stra8*. Half-maximal expression of these genes preceded or coincided with half-maximal expression of *Stra8* (Fig 4D, S5 Fig). In contrast, all five of the Class 3 genes tested reached half-maximal expression after *Stra8* had done so. Thus, the *Stra8*-independent pathway is able to induce expression of some Class 1 and 2 genes in parallel with *Stra8*.

### A *Stra8*-dependent process is required for subsequent down-regulation of *Stra8* and *Rec8*

Spatiotemporal analysis of *Rec8* expression in *Stra8*-deficient germ cells unexpectedly revealed that in the absence of *Stra8*, germ cells expressed *Rec8* at modestly higher levels (Figs 3F and 4E), and *Rec8* expression persisted for at least a day longer than in wild type. Therefore, a *Stra8*-dependent process is required for the subsequent down-regulation of *Rec8*. By our measurements, *Rec8* and *Stra8* are induced and subsequently down-regulated with nearly identical dynamics. Therefore, we wondered if *Stra8* down-regulation also requires *Stra8* function. To measure *Stra8* promoter activity in the *Stra8*-deficient germ cells, we measured expression of a *lacZ* reporter knocked into the endogenous *Stra8* locus [20]. We compared this to *lacZ* expression in *Stra8* heterozygotes, where one functional copy of *Stra8* is present. As with expression of *Rec8*, expression of *lacZ* in the homozygous *Stra8* knockout persisted for at least a day longer than in the heterozygous *Stra8* mouse (Fig 4F, S6 Fig). Thus, we have identified down-regulation of *Stra8* and *Rec8* as a novel *Stra8*-dependent event (Fig 4G).

### After down-regulating *Stra8*, germ cells are refractory to further *Stra8* expression

**Fig 5:**
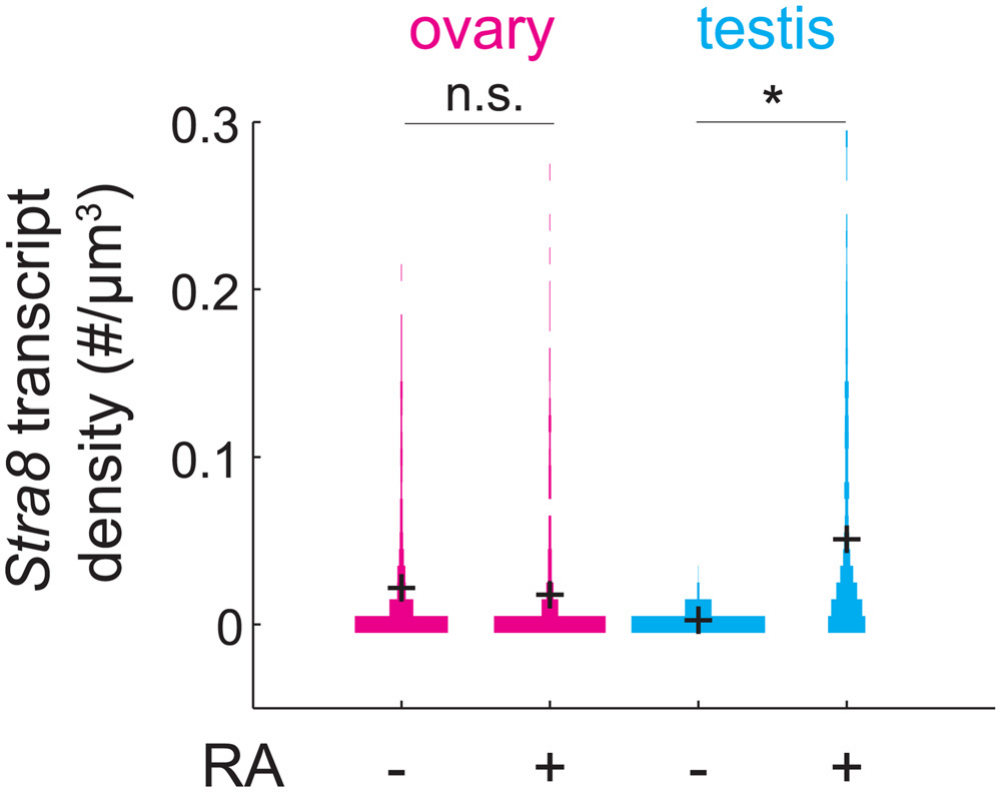
RA is unable to induce *Stra8* in ovarian germ cells that have induced and down-regulated *Stra8*. Distributions of *Stra8* transcript densities in E16.5 ovary (pink) and testis (blue), with and without exogenous RA administered at E15.5. Asterisks represent significant differences between the means of the distributions (p< 0.05, t-test on average transcript densities of biological replicates).

The observation that *Stra8* expression is rapidly down-regulated after its initial induction led us to wonder if, in addition to down-regulating *Stra8*, germ cells become refractory to subsequent induction of *Stra8* by RA. If so, wild-type germ cells that have expressed *Stra8* once should not be able to express *Stra8* again, even if they were provided with a second (exogenous) dose of RA. To test this prediction, we administered exogenous RA to pregnant mice at E15.5, by which time most germ cells have down-regulated *Stra8*. We then measured expression of *Stra8* a day later, at E16.5 (Fig 5). *Stra8* expression was not increased in ovarian germ cells of fetuses that received RA, compared to fetuses that did not receive RA. As a control, we tested if RA was able to induce *Stra8* in E15.5 testicular germ cells. Since testicular germ cells do not ordinarily express *Stra8* until after birth, we expected that they would be able to induce *Stra8* expression if exposed to RA before birth. We found that at E16.5, *Stra8* expression was induced about 20-fold in testicular germ cells of fetuses that received RA, compared to fetuses that did not receive RA. Thus, fetal ovarian germ cells that have down-regulated *Stra8* expression are refractory to re-expressing *Stra8* when exposed to RA 24 hours after initial down-regulation of *Stra8*.

### *Sycp3* induction precedes negative regulation of *Rec8* along the anterior-posterior axis of the E14.5 ovary

**Fig 6:**
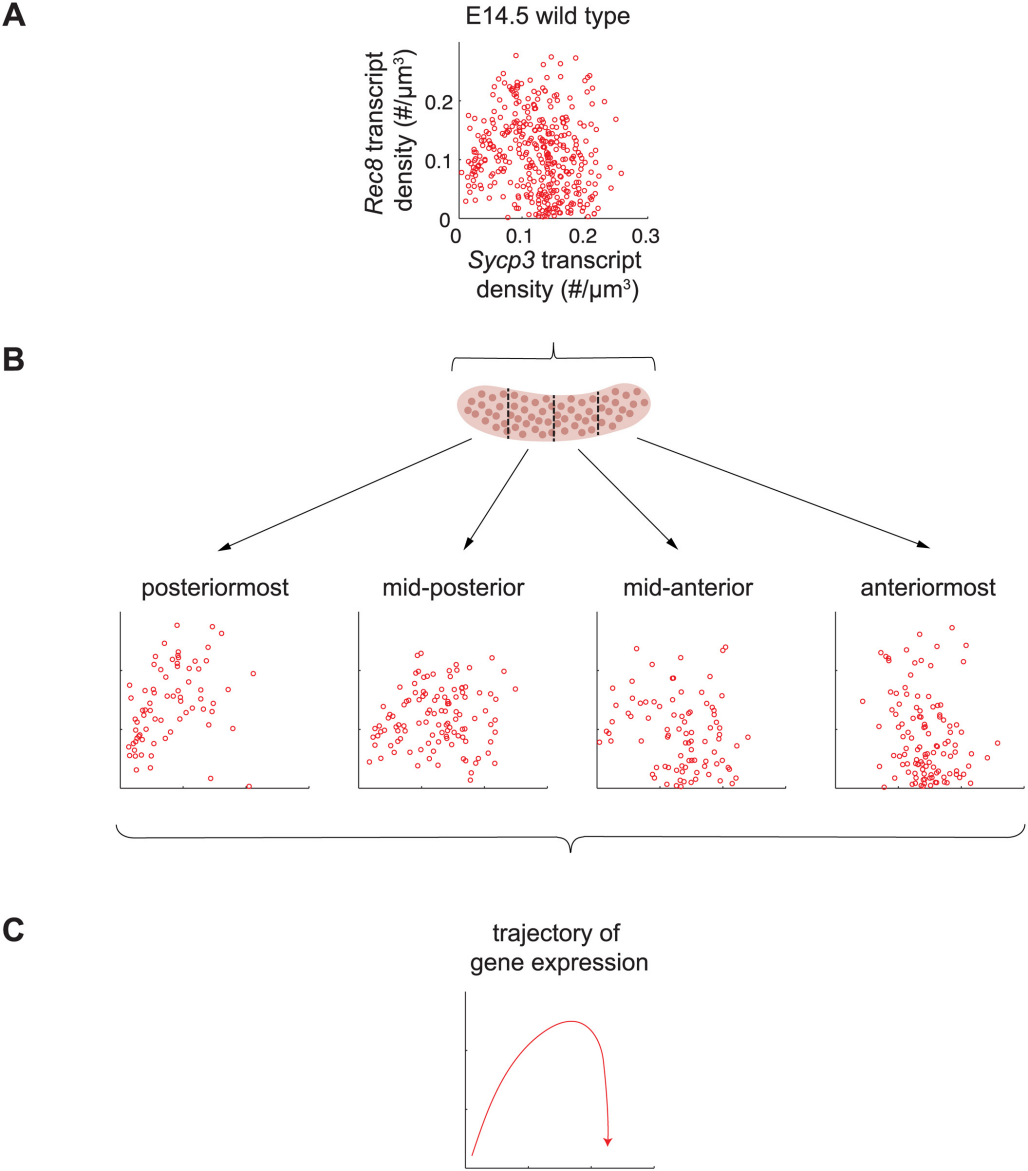
*Sycp3* induction precedes negative regulation of *Rec8* along the anterior-posterior axis of the E14.5 ovary. (A) Representative scatterplot of transcript densities of *Sycp3* against *Rec8* in E14.5 wild-type ovarian germ cells. (B) Scatterplots of transcript densities in germ cells from (A), divided by location in the posteriormost, mid-posterior, mid-anterior, and anteriormost quarters of the ovary. (C) The trajectory of gene expression for an individual germ cell as inferred from gene expression at posterior, middle, and anterior positions.

Earlier, we measured expression of *Rec8* and *Sycp3* in hundreds of individual E14.5 germ cells that lacked *Stra8*, and found that *Rec8* and *Sycp3* levels were positively correlated, implying their co-regulation by a *Stra8*-independent pathway (Fig 3J). We were initially surprised to find, upon performing the same analysis in E14.5 wild-type germ cells (Fig 6A), that *Rec8* and *Sycp3* levels were not correlated in any simple fashion when *Stra8* was present. We reasoned that these differences between wild-type and *Stra8*-deficient germ cells should be due to the two *Stra8*-dependent processes described earlier—partial induction of *Sycp3*, and down-regulation of *Rec8*. Our earlier results suggested that induction of *Sycp3* occurs first, followed by down-regulation of *Rec8*.

To corroborate these understandings, we set out to finely dissect the time course of *Rec8* and *Sycp3* expression by separately analyzing germ cells in posterior-to-anterior quarters of the E14.5 wild-type ovary (Fig 6B). In the posteriormost quarter of the ovary, where germ cells are least differentiated, *Rec8* and *Sycp3* levels were positively correlated (Fig 6B), suggesting that the dominant process at this very early stage is induction of both *Rec8* and *Sycp3*. In subsequent stages, *Sycp3* continues to be induced, but *Rec8* is down-regulated. For example, in the middle quarters of the ovary, where germ cells are more differentiated, many germ cells had increased *Sycp3* levels, but *Rec8* expression was decreased, particularly in those cells with high expression of *Sycp3*. In the anteriormost quarter of the ovary, where germ cell differentiation is most advanced, all germ cells had high *Sycp3* levels, but most had very low levels of *Rec8* expression (Fig 6B). In summary, fetal ovarian germ cells progressed from a state of low *Sycp3*/low *Rec8* expression, to a state of high *Sycp3*/high *Rec8* expression, and finally to a state of high *Sycp3*/low *Rec8* expression (Fig 6C), reflecting initial induction of both *Sycp3* and *Rec8*, and subsequent down-regulation of *Rec8*. As a consequence of down-regulation of *Rec8* (but not *Sycp3*) in the most advanced germ cells, expression of *Rec8* and expression of *Sycp3* were no longer correlated. Thus, the conclusions arising from our earlier analyses were corroborated by positionally informed, single-cell correlation analysis in the E14.5 wild-type ovary.

## Discussion

**Fig 7:**
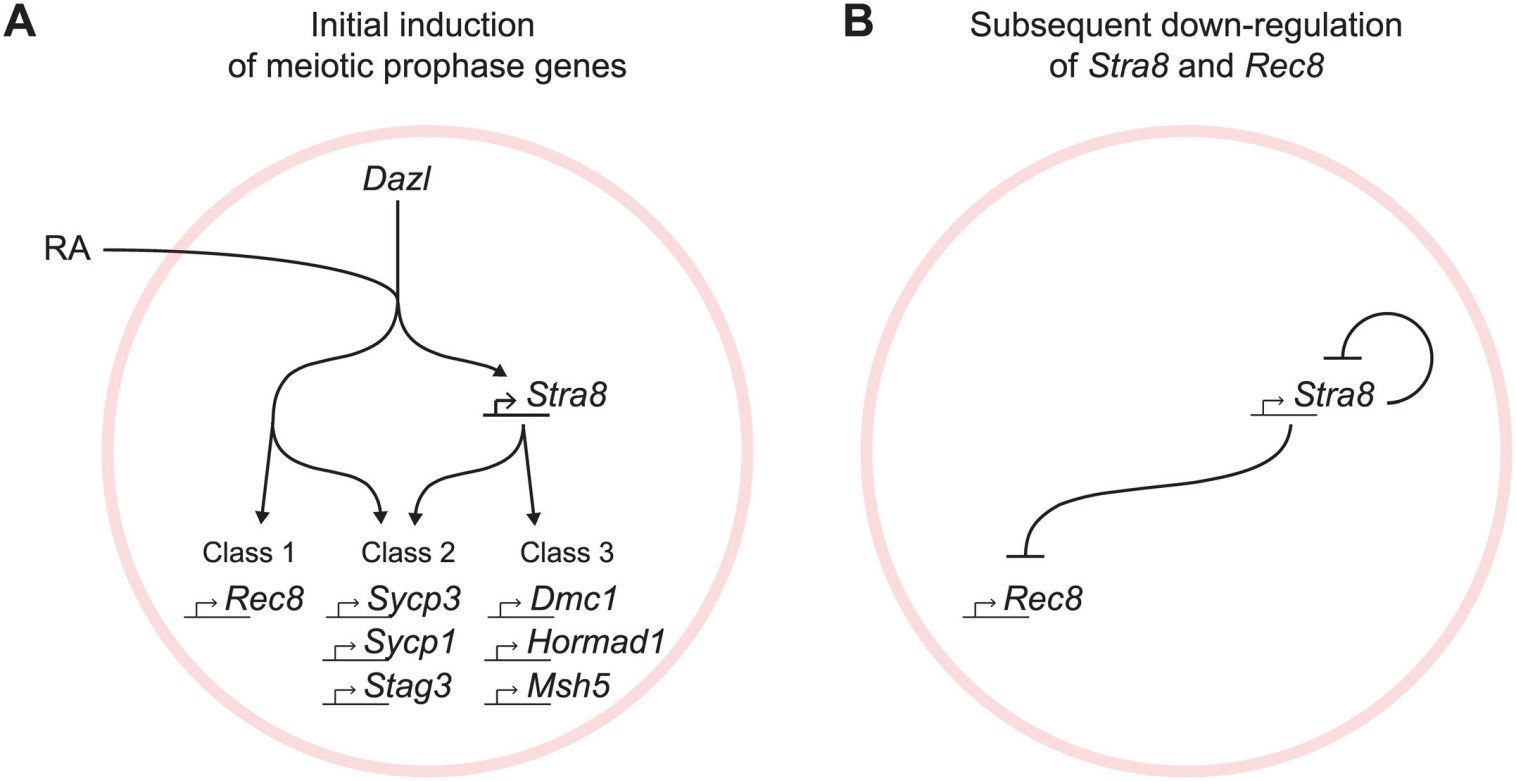
Summary model for induction of meiotic prophase gene expression. (A) Model for initial induction of meiotic prophase genes by *Dazl*, RA, and *Stra8*. In the presence of *Dazl*, RA induces meiotic prophase genes via *Stra8*-independent and *Stra8*-dependent pathways. Genes range across a spectrum of *Stra8*-dependency; for simplicity, we divide the genes into three classes: Class 1 –fully *Stra8*-independent, Class 2 –partially *Stra8*-independent and partially *Stra8*-dependent, and Class 3 –fully *Stra8*-dependent. Representatives of each gene class are shown. (B) A *Stra8*-dependent process is required for down-regulation of *Stra8* and *Rec8* expression.

The regulated induction of meiotic prophase genes is a prerequisite for the chromosomal program of meiotic prophase. We report here a mammalian gene regulatory program for meiotic prophase as it occurs in fetal ovarian germ cells. We identified 104 genes that fulfill stringent criteria for specificity to meiotic prophase. A quarter of these genes have been shown previously to be required for successful meiotic prophase. The remaining three quarters remain uncharacterized and represent promising candidates that may play similarly critical roles during meiotic prophase. Meiotic prophase genes are induced initially by RA, in the presence of *Dazl*, via *Stra8*-independent and *Stra8*-dependent pathways (Fig 7A). Subsequently, down-regulation of *Stra8* and *Rec8* occurs via a *Stra8*-dependent process (Fig 7B). We propose that these two elements of gene regulation enable germ cells to prepare for the chromosomal program of meiotic prophase, and to ensure that the chromosomal program is induced just once.

### Gene induction via *Stra8*-independent and *Stra8*-dependent pathways represents a multi-output feedforward loop that enables a temporal order of gene activation

At the onset of meiotic prophase, meiotic prophase genes are induced by *Dazl*, RA, and *Stra8*, organized in two branching pathways (Fig 7A). *Dazl* is required for the induction of nearly all genes expressed specifically during meiotic prophase. While the results highlight *Dazl*’s crucial role in the gene expression program for meiotic prophase, the mechanism by which it enables gene expression remains unclear. Downstream of *Dazl*, gene induction occurs via a *Stra8*-independent pathway as well as a *Stra8*-dependent pathway; these pathways function both separately and additively. Expression of some genes requires only the *Stra8*-independent pathway (Class 1), while other genes require both *Stra8*-independent and *Stra8*-dependent pathways (Class 2), and yet other genes are fully *Stra8*-dependent (Class 3). Both the *Stra8*-dependent and *Stra8*-independent pathways are regulated by RA.

These genetic insights lead us to two speculative hypotheses regarding molecular mechanisms of meiotic prophase gene regulation by STRA8 and RA receptors (RARs): (1) The *Stra8*-dependent pathway is mediated directly by the STRA8 protein, a putative basic helix-loop-helix transcription factor, and (2) the *Stra8*-independent pathway is mediated directly by RARs. Transcriptome data from both whole gonads (this study) and sorted germ cells [34] support this possibility: fetal ovarian germ cells initiating meiosis express all three RARs (RAR alpha, beta, and gamma) and their heterodimeric partners, the retinoid X receptors (RXR alpha, beta, and gamma). Potential redundancies among the RARs and RXRs complicate genetic interrogation of the roles of the RARs. The possible roles of STRA8 and RARs in directly regulating gene expression can be tested by chromatin-immunoprecipitation-sequencing (ChIP-seq) of RARs and STRA8 in germ cells that are initiating meiosis. We predict that Class 1 genes will be bound by RARs but not STRA8, Class 3 genes will be bound by STRA8 but not RARs, and Class 2 genes will be bound by both RARs and STRA8. A ChIP-Seq study in embryonic stem cells identified RAR binding of both the *Stra8* and *Rec8* promoter regions [35]. Of course, it is also possible that the *Stra8*-independent and *Stra8*-dependent pathways are mediated indirectly, by germ-cell-expressed factors that have not yet been implicated in meiotic initiation.

The branching regulatory model described here is reminiscent of a motif termed a feed forward loop (FFL), which has been shown to generate a temporal order of gene activation [36]. An FFL comprises an upstream regulator, in this case RA, which regulates a downstream regulator, in this case *Stra8*. Both the upstream regulator, RA, and the downstream regulator, *Stra8*, regulate multiple downstream targets, in this case the meiotic prophase genes. Genes respond to input from either the upstream or downstream regulator, or both. Modulating the activation strengths of upstream versus downstream regulators can generate a temporal order of gene activation: genes with greater input from the upstream regulator are activated earlier, and genes with greater input from the downstream regulator are activated later. Consistent with such an outcome, we observe that a subset of Class 1 and 2 genes, which are fully or partially induced by the *Stra8*-independent pathway, are expressed earlier than Class 3 genes and with timing of induction close to that of *Stra8* induction.

We propose that Class 1 and 2 genes may be induced earlier so as to prepare cells for the meiotic chromosomal events triggered by *Stra8*. Indeed, we observe that Class 1 and 2 genes include almost all known meiotic cohesins and synaptonemal complex proteins, which structurally associate with meiotic chromosomes and may therefore be required early, and in sufficient quantities to satisfy the stoichiometric requirements of the chromosomal program. Early expression of cohesin and synaptonemal complex proteins, prior to initiation of the chromosomal program, may be a common feature of both sexes and across species. In mouse testicular germ cells, the synaptonemal complex protein genes *Sycp1*, *Sycp2*, and *Sycp3* are expressed as early as in mitotic spermatogonia [37]. In the *C*. *elegans* gonad and the *D*. *melanogaster* ovary, *Rec8* and synaptonemal complex proteins respectively are also expressed during the amplifying mitotic divisions preceding meiosis [38–40].

Induction of Class 2 genes by a combination of *Stra8*-independent and *Stra8*-dependent pathways may also contribute to fine-tuning expression levels of meiotic prophase genes. Precise regulation of gene dosage has been shown to be important for meiotic chromosomal processes. In the mouse, heterozygous loss of function for either one of the cohesins *Smc1b* and *Rec8* perturbs formation of the synaptonemal complex and affects synapsis and recombination between homologs [41]. Therefore, although the *Stra8*-independent pathway is sufficient for partial gene expression, the two pathways in combination may serve to optimize levels of gene expression and chromosomal function of the Class 2 genes.

### Down-regulation of *Stra8* via a *Stra8*-dependent process may ensure that the chromosomal program is initiated only once in each cell

Subsequent to the initial induction of *Stra8* and *Rec8*, their expression declines rapidly. We discovered that this down-regulation depends on *Stra8*. It remains to be determined whether this occurs directly, via *Stra8* activity as a putative transcriptional regulator, or indirectly, as a consequence of progression of cell state. In either case, we propose that *Stra8*-dependent down-regulation of *Stra8* and *Rec8* may serve to limit gene expression to their appropriate window of function. In particular, *Stra8*-dependent down-regulation of itself represents a negative feedback loop that prevents prolonged induction of the chromosomal program of meiotic prophase. In addition, we found that ovarian germ cells that have down-regulated *Stra8* are refractory to re-expressing *Stra8* even in the presence of exogenous RA, which may prevent re-initiation of the chromosomal program. In yeast, an analogous negative feedback loop is postulated to restrict supernumerary rounds of DNA replication and nuclear division. IME1, a transcription factor that initiates the yeast meiotic transcriptional program, induces IME2, which restricts expression of IME1 and destabilizes IME1 protein [3,42]. Absence of IME2 results in prolonged IME1 expression and additional rounds of DNA synthesis and nuclear division [43].

### Implications for the gene regulatory program of meiotic prophase in the male

Based on similarities between ovarian and testicular germ cells, it is likely that the gene regulatory program as inferred from fetal ovarian germ cells is shared, at least in part, between the sexes. In both sexes, RA induction of *Stra8* has been shown to be required for initiation of the chromosomal program of meiotic prophase [12,14,20]. RA and *Stra8* could therefore also regulate gene expression in the male. In testicular germ cells entering meiosis, *Stra8* is also rapidly induced, at pre-leptotene, and then rapidly down-regulated, by leptotene [17], suggesting the possibility that there is also negative feedback on *Stra8* expression. However, several aspects of regulation in the male remain unclear. For instance, in the male, RA-STRA8 signaling regulates not only meiotic initiation but also spermatogonial differentiation [44]. In fetal ovarian germ cells, competence to respond to RA and initiate meiosis requires *Dazl* [21]; in testicular germ cells, *Dazl*’s role in meiotic competence remains unknown. Thus, studies of adult testicular germ cells deficient for *Dazl* or *Stra8* will be required to determine if *Dazl* and *Stra8* govern the gene regulatory program of meiotic prophase in the adult testis in a manner similar to that reported here for the fetal ovary.

### Implications for in vitro germ cell derivation

Our findings have practical implications for *in vitro* derivation of germ cells and gametes. First, our results provide a blueprint to guide efforts in recapitulating the gene regulatory program of meiotic prophase *in vitro*. Second, our findings substantiate previous criticisms against taking expression of meiotic genes as sufficient evidence of meiosis [45,46]. By explicitly interrogating the regulation of the gene expression program and the chromosomal program by *Dazl* and *Stra8*, we showed that the two programs are regulated distinctly. Specifically, the chromosomal program of meiotic prophase requires *Stra8* function, but a subset of the gene expression program is induced independently of *Stra8*. Our findings thus highlight gene expression as a preparatory phase for the chromosomal program, and underscore the insufficiency of meiotic gene expression as an assay for meiotic progression. Rather, both the gene regulatory program and the chromosomal program are essential for successful meiosis.

## Materials and Methods

### Ethics statement

All experiments involving mice were performed in accordance with the guidelines of the Massachusetts Institute of Technology (MIT) Division of Comparative Medicine, which is overseen by MIT's Institutional Animal Care and Use Committee (IACUC). The animal care program at MIT/Whitehead Institute is accredited by the Association for Assessment and Accreditation of Laboratory Animal Care, International (AAALAC), meeting or exceeding the standards of AAALAC as detailed in the Guide for the Care and Use of Laboratory Animals. The MIT IACUC approved this research (no. 0714-074-17).

### Mice

Germ-cell-depleted (*Kit* ^*W*^ */Kit* ^*Wv*^) and homozygous wild-type control (*Kit* ^+^ */Kit* ^+^) were generated by crossing C57BL/6J-*Kit* ^*Wv*^ */Kit* ^+^ (The Jackson Laboratory) males to WBB6F1/J-*Kit* ^*W*^ */Kit* ^+^ (The Jackson Laboratory) females [47]. *Kit* ^*W*^ and *Kit* ^*Wv*^ alleles were genotyped as previously described [48,49]. *Stra8*-deficient (*Stra8* ^-/-^), *Dazl*-deficient (*Dazl* ^-/-^) and homozygous wild-type control embryos were generated by heterozygote matings of *Dazl* ^*tm1Hjc*^ [50] and *Stra8* ^*tm1Dcp*^ [20] mice respectively. *Dazl* ^*tm1Hjc*^, *Stra8* ^*tm1Dcp*^, and wild-type mice used are of C57BL/6 background. *Dazl* ^*tm1Hjc*^ and *Stra8* ^*tm1Dcp*^ alleles were genotyped as previously described [20,50].

### RA treatment

500 mg/kg of body weight all-trans RA (Sigma-Aldrich, St Louis, MO), dissolved at 30 mg/ml of corn oil, was administered to pregnant mice via gavage.

### Embryonic gonad collection and sexing

Timed matings were set up by housing female mice with male mice overnight. Noon of the day when a vaginal plug was evident was considered E0.5. For RNA-seq analysis, embryonic gonads were dissected away from mesonephroi. For RNA-seq from germ-cell-depleted (*Kit* ^*W*^ */Kit* ^*Wv*^) and homozygous wild-type control (*Kit* ^+^ */Kit* ^+^) gonads, *Stra8*-deficient (*Stra8* ^-/-^), *Dazl*-deficient (*Dazl* ^-/-^) and homozygous wild-type control gonads, whole gonads were processed for sequencing. For RNA-seq from E12.5 and E13.5 anterior and posterior portions of the ovary, ovaries were dissected into thirds, and the anterior and posterior thirds were processed for sequencing. For smFISH, embryonic gonads were dissected with mesonephroi intact to provide anterior-posterior orientation. For embryos E13.5 and older, the sex of tissues was determined by scoring the presence or absence of testicular cords. For E11.5 and E12.5 embryos, sex was determined by PCR as previously described [23].

### RNA-seq sample preparation

For all RNA-seq experiments, total RNA (~1 ug) was extracted from embryonic gonads using Trizol (Invitrogen) according to the manufacturer’s protocol, and hemoglobin transcripts were selectively removed from total RNA using GLOBINclear (Invitrogen, Carlsbad, CA) according to the manufacturer’s protocol. For *Kit* ^*W*^ */Kit* ^*Wv*^ and *Kit* ^+^ */Kit* ^+^ embryonic gonads and E12.5 and E13.5 embryonic ovary thirds, libraries were prepared using the Illumina mRNA-Seq Sample Preparation Kit (Illumina, San Diego, CA) according to the manufacturer’s protocol. Libraries were sequenced on the Illumina Genome Analyzer II platform to obtain 36-base-pair single reads. For E14.5 *Dazl*-deficient, *Stra8*-deficient, and wild-type control ovaries, libraries were prepared using the Illumina TruSeq RNA Sample Preparation Kit. Libraries were multiplexed and sequenced on the Illumina HiSeq 2000 platform to obtain 40-base-pair single reads. RNA-seq data for *Kit* ^*W*^ */Kit* ^*Wv*^ and *Kit* ^+^ */Kit* ^+^, E12.5 and E13.5 embryonic ovary thirds, and E14.5 *Dazl*-deficient, *Stra8*-deficient, and wild-type control ovaries have been deposited in NCBI GEO under accession number GSE70361 and NCBI SRA under accession numbers SRP058992, SRP059594, SRP059601, and SRP059599.

RNA-seq on gonads from *Kit* ^*W*^ */Kit* ^*Wv*^ and *Kit* ^+^ */Kit* ^+^ embryos was performed on two biological replicates for each condition. RNA-seq on anterior and posterior thirds of ovaries from E12.5 and E13.5 wild-type embryos was performed on two and three replicate pools respectively, where each pool consisted of ovary thirds from eight embryos. RNA-seq on E14.5 *Dazl*-deficient and *Stra8*-deficient ovaries was performed on three biological replicates each, with paired homozygous wild-type controls.

### RNA-seq data analysis

Reads were aligned to the mouse genome (mm9) using TopHat [51], allowing only unique alignments (option—g1). We counted reads mapping to the Refseq annotated gene set using htseq-count [52]. Fold-changes and FDR-corrected p values, q, for differentially expressed genes were calculated using edgeR, using tagwise-dispersions and normalizing for library complexity [53]. FPKMs were calculated using Cufflinks [54]. K-means clustering was performed using Cluster 3.0 on log transformed and mean centered FPKMs, using the Pearson correlation as the similarity metric [55], and visualized using Treeview [56]. Non-coding genes were excluded from analyses.

### Single-molecule Fluorescent In Situ Hybridization (smFISH)

Probe design, synthesis, and coupling were as previously described [33,57]. Probes sequences are provided in S2 Text. Gonads were fixed in 4% paraformaldehyde (PFA)/PBS for 2 hours at 4°C, incubated overnight in 30% sucrose/4% PFA/PBS at 4°C, then embedded in O.C.T. compound (Sakura Finetek, Torrance, CA). Frozen blocks were sectioned at 8 μm thickness, fixed in 4% formaldehyde at room temperature for 15 minutes, rinsed in PBS, and dehydrated overnight in 70% ethanol at 4°C. The hybridization procedure was performed as previously described [33,57]. FITC-coupled anti-SSEA-1 antibody (BD 560127) (BD Biosciences, Franklin Lakes, NJ) was added to the hybridization step at 1:30 to identify germ cells. In all experiments, germ cells were identified by either smFISH for *Dazl* or *Oct4*, in combination with SSEA1 immunostaining, and/or DAPI nuclear staining. Counting of individual mRNA particles, image stitching, and data analysis was performed using custom Matlab software as previously described [33,57].

To depict distributions of transcript densities for each group, we pooled biological replicates in one violin plot. Comparison of groups was performed by comparing means of at least two biological replicates from at least two litters using the two-sample t-test. To depict correlations between pairs of genes in individual cells, we show one representative biological replicate, but calculate the Spearman correlation coefficient for each biological replicate. To depict average transcript densities over space and time, we pooled biological replicates. At each time point, we determined average transcript densities from the posterior to anterior of the ovary for 100 windows of size 0.2 of the total length of the ovary. The average transcript density traces of consecutive time points were joined together from posterior to anterior. Using average transcript density traces of *Stra8* as a guide, we overlapped some time points by shifting along the x-axis in order to maximize overlap between the average expression traces for *Stra8*. We determined shifts using *Stra8* expression, and applied the same shifts to spatiotemporal plots for all other genes.

## Supporting Information

S1 Fig *Rec8* is expressed in both somatic and germ cells. E14.5 fetal ovaries stained with DAPI, and for SSEA1 (immunofluorescence), *Rec8* (single molecule fluorescent in situ hybridization, smFISH), and *Dazl* (smFISH). Single transcripts of *Rec8* and *Dazl* are detected as punctate signals by smFISH. Germ cells are outlined in red.
(EPS)

S2 Fig Transcript densities of meiotic prophase genes as measured by single molecule FISH. (A) Violin plots representing distribution of transcript densities in wild type (red), *Stra8* mutant (blue) and *Dazl* mutant (green), for 13 selected meiotic prophase genes. Asterisks represent significant differences between the means of distributions (t-test on average transcript density of a population of germ cells from independent biological replicates). n.s. denotes differences that are not statistically significant (p > 0.05). For *Mei1* and *Msh5*, expression in *Stra8* and *Dazl* mutants averaged less than 1 transcript per cell, so differences between *Stra8* and *Dazl* mutants were not tested. Genes were grouped into class 1, 2, and 3 based on significant differences in expression between wild-type, *Stra8*-deficient, and *Dazl*-deficient germ cells as described in main text. *Sycp2* transcript densities do not differ significantly between wild-type and *Stra8*-deficient germ cells. Nevertheless, we placed it in class 2 based on subsequent analyses that show that by E15.5, it is expressed at significantly lower levels in *Stra8*-deficient compared to wild-type germ cells (S3 Fig, p = 0.0088 at E15.5). (B) Scatterplot of log2 fold-change of gene expression in E14.5 *Stra8* mutant over wild-type ovary against *Dazl* mutant over wild-type ovary for 104 meiotic prophase genes, as measured by RNA-seq. Thirteen selected meiotic prophase genes that were also examined by smFISH are highlighted in color. Red, orange, and yellow represent class 1, 2, and 3 genes respectively, as defined by smFISH.
(EPS)

S3 Fig Single cell correlation of expression of meiotic prophase genes compared to *Rec8* in *Stra8*-deficient ovaries. Representative scatterplots of transcript densities of 12 meiotic prophase genes from class 2 and 3 (y-axis) against *Rec8* (x-axis) as measured in E14.5 *Stra8*-deficient ovaries. Correlation coefficients for both plots shown, and for biological replicates, are given in S3 Table.
(EPS)

S4 Fig Correlation of change over time versus anterior-posterior position. Scatterplot of log2 fold-change in expression of 527 ovarian germ-cell-enriched genes in time (E13.5 anterior over E12.5 anterior) versus space (E13.5 anterior over E13.5 posterior). Source data is provided in S4 Table.
(EPS)

S5 Fig Spatiotemporal analyses of gene expression by single molecule FISH. Spatiotemporal plot of average transcript densities of class 2 and 3 genes along anterior-posterior axis of the ovary at E11.5, E12.5, E13.5, E14.0, E14.5, E15.0, E15.5, and E16.5. (*Rec8* and *Sycp3* are shown in Fig 4.)
(EPS)

S6 Fig Single molecule FISH analyses of *Stra8* and *Stra8*-*lacZ* reporter. Representative scatterplot of transcript densities of *Stra8*-*lacZ* reporter (y-axis) against *Stra8* (x-axis) from E14.5 *Stra8* wild type/*lacZ* heterozygote, showing correlation of *lacZ* and *Stra8* expression.
(EPS)

S1 Table Expression, and differential expression, of 527 ovarian germ-cell-enriched genes, as well as all Refseq annotated genes, in E12.5, E14.5, and E16.5 in wild-type (*Kit* ^+^/*Kit* ^+^) and germ-cell-depleted (*Kit* ^*W*^/*Kit* ^*Wv*^) ovary and testis. (XLSX)

S2 Table Expression, and differential expression, of 104 meiotic prophase-specific genes, as well as all Refseq annotated genes, in E14.5 wild-type, *Stra8*-deficient, and *Dazl*-deficient ovary. (XLSX)

S3 Table Correlation coefficients for expression of meiotic prophase genes against *Rec8*. (XLSX)

S4 Table Expression, and differential expression, of 527 ovarian germ-cell-enriched genes in anterior and posterior thirds of E12.5 and E13.5 wild-type ovary. (XLSX)

S1 Text Published data on fertility and/or meiotic defects for 104 meiotic prophase-specific genes. (PDF)

S2 Text smFISH probe sequences. (TXT)
