# Supplementary material for: A Gene Regulatory Program for Meiotic Prophase in the Fetal Ovary
Source: PLoS Genet. 2015 Sep 17;11(9):e1005531. doi: 10.1371/journal.pgen.1005531 (PMC4574967; doi:10.1371/journal.pgen.1005531)
Supplement: S1 Text — (PDF) [file pgen.1005531.s011.pdf]

S1 Text

Number of genes for which a mutant has been analyzed, and a meiotic or fertility defect found: 32/33

Number of genes for which expression pattern has been characterized (in detail, not by microarray or next-generation sequencing screens), and expression corresponding to meiotic prophase found: 53

	Gene	Mutant analyzed	Defect in meiotic prophase	Expression pattern characterized	Expression corresponds to meiotic prophase	References
1	1700013H16Rik (Xlr6)	No		Yes	Yes, from leptotene to pachytene. Contains Cor1 domain.	(1)
2	4930528F23Rik (Meiob)	Yes	Yes	Yes	Yes	(2–4)
3	Asf1b	Not examined in germ cells			Yes	(5)
4	Ccdc155	No		Yes	Yes	(6)
5	Ccnb3	No		Yes	Yes	(7)
6	Cyld	Yes	Spermatogenesis defects, meiotic prophase not characterized	No		(8)
7	D1Pas1	No		Yes	Yes	(9,10)
8	M1ap (D6Mm5e)	Yes	Yes	Yes	Yes	(11,12)
9	Ddb2	Yes	Fertile, but with defects in apoptosis in spermatogenic cells	No	No	(13)
10	Dmc1	Yes	Yes	Yes	Yes	(14,15)
11	Dmrtc2	Yes	Yes, male only	Yes	Yes	(16)
12	Ecsit	Not examined		Yes	Yes	(3)

		in germ cells				
13	Figla	Yes	Defect in ovarian follicle formation	Yes	Yes, but persists beyond meiotic prophase	(17)
14	Fmr1nb	No		Yes	Yes	(3)
15	Gpat2	No		Yes	Most likely	(18)
16	Gpr19	No		Yes	Most likely	(19)
17	Hfm1	Yes	Yes	No		(20)
18	Hormad1	Yes	Yes	Yes	Yes	(21–23)
19	Hormad2	Yes	Yes	Yes	Yes	(24,25)
20	Hsf2bp	No		Yes	Yes	(3)
21	Il18	Not examined in germ cells		Yes	Claimed but data unclear	(26)
22	Inca1	Yes	No. KO mice were viable and fertile.	Yes	Yes	(3)
23	Mei1	Yes	Yes	Yes	Not examined with cellular resolution	(27,28)
24	Msh4	Yes	Yes	Yes	Yes	(30)
25	Msh5	Yes	Yes	No		(31,32)
26	Mtl5	No		Yes	Yes	(33–35)
27	Poln	No		Yes	Yes	(36)
28	Prdm9	Yes	Yes	Yes	Yes	(37–41)
29	Rad21l	Yes	Yes	Yes	Yes	(42–45)
30	Rec8	Yes	Yes	Yes	Yes	(46–48)
31	Rhox13	No		Yes	Yes	(3,49)
32	Setdb2	No		Yes	Yes	(50)
33	Slc25a31	Yes	Yes	Yes	Yes	(51,52)
34	Smc1b	Yes	Yes	Yes	Yes	(53–56)
35	Spata22	Yes	Yes	Yes	Yes	(57)
36	Spata5	No		Yes	Yes	(58)

37	Spdya	No		Yes	Yes	(3)
38	Spo11	Yes	Yes	Yes	Yes	(59–62)
39	Stag3	No		Yes	Yes	(63,64)
40	Stra8	Yes	Yes, both	Yes	Yes	(65–67)
41	Syce1	Yes	Yes, both	Yes	Yes	(68,69)
42	Syce3	Yes	Yes, both	Yes	Yes	(70)
43	Sycp1	Yes	Yes, both	Yes	Yes	(71,72)
44	Sycp2	Yes	Yes, in males, females are subfertile	Yes	Yes	(73,74)
45	Sycp3	Yes	Yes, in males, females are subfertile	Yes	Yes	(75–78)
46	Syn2	Not examined in germ cells		Yes	Yes in fetal ovary and testis	(3)
47	Taf7l	Yes	Males are subfertile with post-meiotic defects, females are fertile	Yes	Yes, in adult testis	(79,80)
47	Tex101	Yes	Males have post-meiotic defects, females are fertile	Yes	Yes	(81,82)
48	Tex11	Yes	Yes, in males, females are subfertile	Yes	Yes, both	(83,84)
49	Tex12	Yes	Yes	Yes	Yes	(85,86)
50	Tex15	Yes	Yes, male only, female are fertile	Yes	Yes	(87,88)
51	Tsc22d3	Yes	Yes, male	Yes	Yes, male	(89–

			only (may be spermatogonial differentiation defect?), female fertile.			91)
52	Tsga10	No		Yes	Yes, male	(92,93)
53	Wbp2nl	No		Yes	Yes, male.	(94)
54	Zfp541	No		Yes	Yes, male	(95)
56	1700011F14Rik (Ccdc172)	No		No		
57	1700123I01Rik	No		No		
58	3110057O12Rik	No		No		
59	4930432K21Rik	No		No		
60	4930447C04Rik	No		No		
61	4930455F23Rik (Ccdc181)	No		No		
62	4930524B15Rik	No		No		
63	4933416C03Rik	No		No		
64	4933427D06Rik	No		No		
65	Adarb1	Not examined in germ cells		No		
66	Aspa	Not examined in germ cells		No		
67	BC049762	No		No		
68	BC051142	No		No		
69	Caprin2	No		Not examined in germ cells		
70	Ccdc36	No		No		

71	Ccdc73	No		No		
72	Ccdc79	No		No		
73	Cdkl2	No		Not examined in germ cells		
74	Cntd1	No		No		
75	Crebl2	No		No		
76	Dennd4a	No		No		
77	Dopey1	No		No		
78	Eaf2	Not examined in germ cells		No		
79	Fbxo47	No		No		
80	Fhl4	No		No		
81	Gm13718	No		No		
82	Gm1564	No		No		
83	H2-D1	Not examined in germ cells		No		
84	H2-K1	Not examined in germ cells		No		
85	Haus8	No		No		
86	Larp1b	No		No		
87	Lypd4	No		No		
88	Madd	Not examined in germ cells		No		
89	Pet2	No		No		
90	Phka2	No		No		
91	Pparg	No		No		
92	Pramel1	No		No		
93	Rad51ap2	No		No		
94	Rbpms2	No		No		
95	Reg2	No		No		
96	Ribc1	No		No		
97	Spryd3	No		No		
98	Syngr4	No		No		
99	Taf9b	No		No		
100	Tex16	No		No		

101	Tktl1	Not examined in germ cells		No		
102	Ugt8a	Not examined in germ cells		No		
103	Usp32	No		No		
104	Zcwpw1	No		No		

As previously noted, many knock-out phenotypes are sexually dimorphic – males are often infertile and display arrests in meiosis, whereas females are fertile or subfertile (96).

References

1. Tsutsumi M, Kogo H, Kowa-Sugiyama H, Inagaki H, Ohye T, Kurahashi H. Characterization of a novel mouse gene encoding an SYCP3-like protein that relocalizes from the XY body to the nucleolus during prophase of male meiosis I. Biol Reprod. 2011;85:165–71. doi:10.1095/biolreprod.110.087270

2. Kogo H, Kowa-Sugiyama H, Yamada K, Bolor H, Tsutsumi M, Ohye T, et al. Screening of genes involved in chromosome segregation during meiosis I: toward the identification of genes responsible for infertility in humans. J Hum Genet. Nature Publishing Group; 2010;55:293–9. doi:10.1038/jhg.2010.26

3. McMahon AP, Aronow BJ, Davidson DR, Davies JA, Gaido KW, Grimmond S, et al. GUDMAP: the genitourinary developmental molecular anatomy project. J Am Soc Nephrol. 2008;19:667–71. doi:10.1681/ASN.2007101078

4. Souquet B, Abby E, Hervé R, Finsterbusch F, Tourpin S, Le Bouffant R, et al. MEIOB Targets Single-Strand DNA and Is Necessary for Meiotic Recombination. PLoS Genet. 2013;9:e1003784. doi:10.1371/journal.pgen.1003784

5. Umehara T, Horikoshi M. Transcription initiation factor IID-interactive histone chaperone CIA-II implicated in mammalian spermatogenesis. J Biol Chem. 2003;278:35660–7. doi:10.1074/jbc.M303549200

6. Morimoto A, Shibuya H, Zhu X, Kim J, Ishiguro K, Han M, et al. A conserved KASH domain protein associates with telomeres, SUN1, and dynactin during mammalian meiosis. J Cell Biol. 2012;198:165–72. doi:10.1083/jcb.201204085

7. Nguyen TB, Manova K, Capodieci P, Lindon C, Bottega S, Wang X-Y, et al. Characterization and expression of mammalian cyclin b3, a prepachytene meiotic cyclin. J Biol Chem. 2002;277:41960–9. doi:10.1074/jbc.M203951200

8. Wright A, Reiley WW, Chang M, Jin W, Lee AJ, Zhang M, et al. Regulation of early wave of germ cell apoptosis and spermatogenesis by deubiquitinating enzyme CYLD. Dev Cell. 2007;13:705–16. doi:10.1016/j.devcel.2007.09.007

9. Session DR, Lee GS, Wolgemuth DJ. Characterization of D1Pas1, a mouse autosomal homologue of the human AZFa region DBY, as a nuclear protein in spermatogenic cells. Fertil Steril. 2001;76:804–11. Available: http://www.ncbi.nlm.nih.gov/pubmed/11591417

10. Vong QP, Li Y, Lau Y-FC, Dym M, Rennert OM, Chan W-Y. Structural characterization and expression studies of Dby and its homologs in the mouse. J Androl. 2006;27:653–61. doi:10.2164/jandrol.106.000471

11. Arango N, Li L, Dabir D. Meiosis I Arrest Abnormalities Lead to Severe Oligozoospermia in Meiosis 1 Arresting Protein (M1ap)-Deficient Mice 1. Biol …. 2012; doi:10.1095/biolreprod.111.098673

12. Arango N a, Huang TT, Fujino A, Pieretti-Vanmarcke R, Donahoe PK. Expression analysis and evolutionary conservation of the mouse germ cell-specific D6Mm5e gene. Dev Dyn. 2006;235:2613–9. doi:10.1002/dvdy.20907

13. Itoh T, Iwashita S, Cohen MB, Meyerholz DK, Linn S. Ddb2 is a haploinsufficient tumor suppressor and controls spontaneous germ cell apoptosis. Hum Mol Genet. 2007;16:1578–86. doi:10.1093/hmg/ddm107

14. Pittman DL, Cobb J, Schimenti KJ, Wilson LA, Cooper DM, Brignull E, et al. Meiotic Prophase Arrest with Failure of Chromosome Synapsis in Mice Deficient for Dmc1 , a Germline-Specific RecA Homolog. 1998;1:697–705.

15. Yoshida K, Kondoh G, Matsuda Y. The Mouse RecA-like Gene Dmc1 Is Required for Homologous Chromosome Synapsis during Meiosis. 1998;1:707–718.

16. Kim S, Namekawa SH, Niswander LM, Ward JO, Lee JT, Bardwell VJ, et al. A mammal-specific Doublesex homolog associates with male sex chromatin and is required for male meiosis. PLoS Genet. 2007;3:e62. doi:10.1371/journal.pgen.0030062

17. Soyal SM, Amleh a, Dean J. FIGalpha, a germ cell-specific transcription factor required for ovarian follicle formation. Development. 2000;127:4645–54. Available: http://www.ncbi.nlm.nih.gov/pubmed/11023867

18. Cattaneo ER, Pellon-Maison M, Rabassa ME, Lacunza E, Coleman R a, Gonzalez-Baro MR. Glycerol-3-phosphate acyltransferase-2 is expressed in spermatic germ cells and incorporates arachidonic acid into triacylglycerols. PLoS One. 2012;7:e42986. doi:10.1371/journal.pone.0042986

19. Hoffmeister-Ullerich S a H, Süsens U, Schaller HC. The orphan G-protein-coupled receptor GPR19 is expressed predominantly in neuronal cells during mouse embryogenesis. Cell Tissue Res. 2004;318:459–63. doi:10.1007/s00441-004-0948-9

20. Guiraldelli MF, Eyster C, Wilkerson JL, Dresser ME, Pezza RJ. Mouse HFM1/Mer3 is required for crossover formation and complete synapsis of homologous chromosomes during meiosis. PLoS Genet. 2013;9:e1003383. doi:10.1371/journal.pgen.1003383

21. Shin Y, Choi Y, Erdin SU, Yatsenko SA, Kloc M, Yang F, et al. Hormad1 Mutation Disrupts Synaptonemal Complex Formation , Recombination , and Chromosome Segregation in Mammalian Meiosis. 2010;6. doi:10.1371/journal.pgen.1001190

22. Daniel K, Lange J, Hached K, Fu J, Anastassiadis K, Roig I, et al. Meiotic homologue alignment and its quality surveillance are controlled by mouse HORMAD1. Nat Cell Biol. Nature Publishing Group; 2011;13:599–610. doi:10.1038/ncb2213

23. Kogo H, Tsutsumi M, Ohye T, Inagaki H, Abe T, Kurahashi H. HORMAD1-dependent checkpoint/surveillance mechanism eliminates asynaptic oocytes. Genes Cells. 2012;17:439–54. doi:10.1111/j.1365-2443.2012.01600.x

24. Wojtasz L, Cloutier JM, Baumann M, Daniel K, Varga J, Fu J, et al. Meiotic DNA double-strand breaks and chromosome asynapsis in mice are monitored by distinct HORMAD2-independent and -dependent mechanisms. Genes Dev. 2012;26:958–73. doi:10.1101/gad.187559.112

25. Kogo H, Tsutsumi M, Inagaki H, Ohye T, Kiyonari H, Kurahashi H. HORMAD2 is essential for synapsis surveillance during meiotic prophase via the recruitment of ATR activity. Genes Cells. 2012;17:897–912. doi:10.1111/gtc.12005

26. Strand M-L, Wahlgren A, Svechnikov K, Zetterström C, Setchell BP, Söder O. Interleukin-18 is expressed in rat testis and may promote germ cell growth. Mol Cell Endocrinol. 2005;240:64–73. doi:10.1016/j.mce.2005.05.011

27. Libby BJ, Reinholdt LG, Schimenti JC. Positional cloning and characterization of Mei1, a vertebrate-specific gene required for normal meiotic chromosome synapsis in mice. Proc Natl Acad Sci. 2003;100:15706–11. doi:10.1073/pnas.2432067100

28. Libby BJ, Fuente RD La, Brien MJO, Wigglesworth K, Cobb J, Inselman A, et al. The Mouse Meiotic Mutation mei1 Disrupts Chromosome Synapsis with Sexually Dimorphic Consequences for Meiotic Progression. 2002;187:174–187. doi:10.1006/dbio.2001.0535

29. Björk JK, Sandqvist A, Elsing AN, Kotaja N, Sistonen L. miR-18, a member of Oncomir-1, targets heat shock transcription factor 2 in spermatogenesis. Development. 2010;137:3177–84. doi:10.1242/dev.050955

30. Kneitz B, Cohen PE, Avdievich E, Zhu L, Kane MF, Hou H, et al. MutS homolog 4 localization to meiotic chromosomes is required for chromosome pairing during meiosis in male and female mice. 2000;1085–1097. doi:10.1101/gad.14.9.1085

31. Vries SS De, Baart EB, Dekker M, Siezen A, Rooij DG De, Boer P De, et al. Mouse MutS-like protein Msh5 is required for proper chromosome synapsis in male and female meiosis. 1999;523–531.

32. Edelmann W, Cohen PE, Kneitz B, Winand N, Lia M, Heyer J, et al. Mammalian MutS homologue 5 is required for chromosome pairing in meiosis. 1999;21:123–127.

33. Olesen C, Møller M, Byskov AG. Tesmin transcription is regulated differently during male and female meiosis. Mol Reprod Dev. 2004;67:116–26. doi:10.1002/mrd.20007

34. Matsuura T, Kawasaki Y, Miwa K, Sutou S, Ohinata Y, Yoshida F, et al. Germ cell-specific nucleocytoplasmic shuttling protein, tesmin, responsive to heavy metal stress in mouse testes. J Inorg Biochem. 2002;88:183–91. Available: http://www.ncbi.nlm.nih.gov/pubmed/11803038

35. Sutou S, Miwa K, Matsuura T, Kawasaki Y, Ohinata Y, Mitsui Y. Native tesmin is a 60-kilodalton protein that undergoes dynamic changes in its localization during spermatogenesis in mice. Biol Reprod. 2003;68:1861–9. doi:10.1095/biolreprod.102.005603

36. Marini F, Kim N, Schuffert A, Wood RD. POLN, a nuclear PolA family DNA polymerase homologous to the DNA cross-link sensitivity protein Mus308. J Biol Chem. 2003;278:32014–9. doi:10.1074/jbc.M305646200

37. Hayashi K, Yoshida K, Matsui Y. A histone H3 methyltransferase controls epigenetic events required for meiotic prophase. Nature. 2005;438:1–4. doi:10.1038/nature04112

38. Mihola O, Trachtulec Z, Vlcek C, Schimenti J, Forejt J. A mouse speciation gene encodes a meiotic histone H3 methyltransferase. Science (80-). 2009;323:373–375. Available: http://www.sciencemag.org/content/323/5912/373.short

39. Baudat F, Buard J, Grey C, Fledel-Alon A. PRDM9 is a major determinant of meiotic recombination hotspots in humans and mice. Science (80-). 2010; Available: http://www.sciencemag.org/content/327/5967/836.short

40. Parvanov E, Petkov P, Paigen K. Prdm9 controls activation of mammalian recombination hotspots. Science (80-). 2010;2010. Available: http://www.sciencemag.org/content/327/5967/835.short

41. Grey C, Barthès P, Chauveau-Le Friec G, Langa F, Baudat F, de Massy B. Mouse PRDM9 DNA-binding specificity determines sites of histone H3 lysine 4 trimethylation for initiation of meiotic recombination. PLoS Biol. 2011;9:e1001176. doi:10.1371/journal.pbio.1001176

42. Lee J, Hirano T. RAD21L, a novel cohesin subunit implicated in linking homologous chromosomes in mammalian meiosis. J Cell Biol. 2011;192:263–76. doi:10.1083/jcb.201008005

43. Herrán Y, Gutiérrez-Caballero C, Sánchez-Martín M, Hernández T, Viera A, Barbero JL, et al. The cohesin subunit RAD21L functions in meiotic synapsis and exhibits sexual dimorphism in fertility. EMBO J. 2011;30:3091–105. doi:10.1038/emboj.2011.222

44. Gutiérrez-caballero C, Herrán Y, Sánchez-Martín M, Suja JÁ, Barbero JL, Alberto M, et al. Identification and molecular characterization of the mammalian α-kleisin RAD21L. Cell …. 2011;10:1477–1487. doi:10.4161/cc.10.9.15515

45. Ishiguro K, Kim J, Fujiyama-Nakamura S, Kato S, Watanabe Y. A new meiosis-specific cohesin complex implicated in the cohesin code for homologous pairing. EMBO Rep. Nature Publishing Group; 2011;12:267–75. doi:10.1038/embor.2011.2

46. Xu H, Beasley MD, Warren WD, van der Horst GTJ, McKay MJ, Horst GTJ Van Der. Absence of mouse REC8 cohesin promotes synapsis of sister chromatids in meiosis. Dev Cell. 2005;8:949–61. doi:10.1016/j.devcel.2005.03.018

47. Bannister LA, Reinholdt LG, Munroe RJ, Schimenti JC. Positional cloning and characterization of mouse mei8, a disrupted allele of the meiotic cohesin Rec8. Genesis. 2004;40:184–94. doi:10.1002/gene.20085

48. Lee J, Iwai T, Yokota T, Yamashita M. Temporally and spatially selective loss of Rec8 protein from meiotic chromosomes during mammalian meiosis. J Cell Sci. 2003;116:2781–90. doi:10.1242/jcs.00495

49. Geyer CB, Saba R, Kato Y, Anderson AJ, Chappell VK, Saga Y, et al. Rhox13 is translated in premeiotic germ cells in male and female mice and is regulated by NANOS2 in the male. Biol Reprod. 2012;86:127. doi:10.1095/biolreprod.111.094938

50. Hogarth C a, Mitchell D, Evanoff R, Small C, Griswold M. Identification and expression of potential regulators of the mammalian mitotic-to-meiotic transition. Biol Reprod. 2011;84:34–42. doi:10.1095/biolreprod.110.086215

51. Brower J V, Lim CH, Jorgensen M, Oh SP, Terada N. Adenine nucleotide translocase 4 deficiency leads to early meiotic arrest of murine male germ cells. Reproduction. 2009;138:463–70. doi:10.1530/REP-09-0201

52. Brower J V, Rodic N, Seki T, Jorgensen M, Fliess N, Yachnis AT, et al. Evolutionarily conserved mammalian adenine nucleotide translocase 4 is essential for spermatogenesis. J Biol Chem. 2007;282:29658–66. doi:10.1074/jbc.M704386200

53. Revenkova E, Eijpe M, Heyting C, Hodges CA, Hunt PA, Liebe B, et al. Cohesin SMC1β is required for meiotic chromosome dynamics , sister chromatid cohesion and DNA recombination. 2004;6:555–562. doi:10.1038/ncb1135

54. Novak I, Wang H, Revenkova E, Jessberger R, Scherthan H, Höög C. Cohesin Smc1beta determines meiotic chromatin axis loop organization. J Cell Biol. 2008;180:83–90. doi:10.1083/jcb.200706136

55. Hodges C a, Revenkova E, Jessberger R, Hassold TJ, Hunt P a. SMC1beta-deficient female mice provide evidence that cohesins are a missing link in age-related nondisjunction. Nat Genet. 2005;37:1351–5. doi:10.1038/ng1672

56. Revenkova E, Eijpe M, Heyting C. Novel meiosis-specific isoform of mammalian SMC1. Mol Cell Biol. 2001;21:6984–6998. doi:10.1128/MCB.21.20.6984

57. La Salle S, Palmer K, O'Brien M, Schimenti JC, Eppig J, Handel MA. Spata22, a novel vertebrate-specific gene, is required for meiotic progress in mouse germ cells. Biol Reprod. 2012;86:45. doi:10.1095/biolreprod.111.095752

58. Liu Y, Black J, Kisiel N, Kulesz-Martin MF. SPAF, a new AAA-protein specific to early spermatogenesis and malignant conversion. Oncogene. 2000;19:1579–88. doi:10.1038/sj.onc.1203442

59. Baudat F, Manova K, Yuen JP, Jasin M, Keeney S. Chromosome synapsis defects and sexually dimorphic meiotic progression in mice lacking Spo11. Mol Cell. 2000;6:989–98. Available: http://www.ncbi.nlm.nih.gov/pubmed/11106739

60. Keeney S, Baudat F, Angeles M, Zhou ZH, Copeland NG, Jenkins N a, et al. A mouse homolog of the Saccharomyces cerevisiae meiotic recombination DNA transesterase Spo11p. Genomics. 1999;61:170–82. doi:10.1006/geno.1999.5956

61. Romanienko P, Camerini-Otero R. Cloning, Characterization, and Localization of Mouse and Human SPO11. Genomics. 1999;169:156–169. Available: http://www.sciencedirect.com/science/article/pii/S0888754399959554

62. Romanienko PJ, Camerini-Otero RD. The mouse Spo11 gene is required for meiotic chromosome synapsis. Mol Cell. 2000;6:975–87. Available: http://www.ncbi.nlm.nih.gov/pubmed/11106738

63. Pezzi N, Prieto I, Kremer L, Pérez Jurado L a, Valero C, Del Mazo J, et al. STAG3, a novel gene encoding a protein involved in meiotic chromosome pairing and location of STAG3-related genes flanking the Williams-Beuren syndrome deletion. FASEB J. 2000;14:581–92. Available: http://www.ncbi.nlm.nih.gov/pubmed/10698974

64. Prieto I, Suja J a, Pezzi N, Kremer L, Martínez-A C, Rufas JS, et al. Mammalian STAG3 is a cohesin specific to sister chromatid arms in meiosis I. Nat Cell Biol. 2001;3:761–6. doi:10.1038/35087082

65. Baltus AE, Menke DB, Hu Y-C, Goodheart ML, Carpenter AE, de Rooij DG, et al. In germ cells of mouse embryonic ovaries, the decision to enter meiosis precedes premeiotic DNA replication. Nat Genet. 2006;38:1430–4. doi:10.1038/ng1919

66. Anderson EL, Baltus AE, Roepers-Gajadien HL, Hassold TJ, de Rooij DG, van Pelt AMM, et al. Stra8 and its inducer, retinoic acid, regulate meiotic initiation in both spermatogenesis and oogenesis in mice. Proc Natl Acad Sci USA. 2008;105:14976–80. doi:10.1073/pnas.0807297105

67. Menke DB, Koubova J, Page DC. Sexual differentiation of germ cells in XX mouse gonads occurs in an anterior-to-posterior wave. Dev Biol. 2003;262:303–312. doi:10.1016/S0012-1606(03)00391-9

68. Costa Y, Speed R, Ollinger R, Alsheimer M, Semple C a, Gautier P, et al. Two novel proteins recruited by synaptonemal complex protein 1 (SYCP1) are at the centre of meiosis. J Cell Sci. 2005;118:2755–62. doi:10.1242/jcs.02402

69. Bolcun-Filas E, Hall E, Speed R, Taggart M, Grey C, de Massy B, et al. Mutation of the mouse Syce1 gene disrupts synapsis and suggests a link between synaptonemal complex structural components and DNA repair. PLoS Genet. 2009;5:e1000393. doi:10.1371/journal.pgen.1000393

70. Schramm S, Fraune J, Naumann R, Hernandez-Hernandez A, Höög C, Cooke HJ, et al. A novel mouse synaptonemal complex protein is essential for loading of central element proteins, recombination, and fertility. PLoS Genet. 2011;7:e1002088. doi:10.1371/journal.pgen.1002088

71. Meuwissen RLJ, Offenberg HH, Dietrich AJJ, Riesewijk a, van Iersel M, Heyting C, et al. A coiled-coil related protein specific for synapsed regions of meiotic prophase chromosomes. EMBO J. 1992;11:5091–100. Available: http://www.pubmedcentral.nih.gov/articlerender.fcgi?artid=556987&tool=pmcentrez&rendertype=abstract

72. De Vries F a T, de Boer E, van den Bosch M, Baarends WM, Ooms M, Yuan L, et al. Mouse Sycp1 functions in synaptonemal complex assembly, meiotic recombination, and XY body formation. Genes Dev. 2005;19:1376–89. doi:10.1101/gad.329705

73. Offenberg HH, Schalk JAC, Meuwissen RLJ, Aalderen M Van, Kester HA, Dietrich AJJ, et al. SCP2: a major protein component of the axial elements of synaptonemal complexes of the rat. Nucleic Acids Res. 1998;26:2572–9. Available: http://www.pubmedcentral.nih.gov/articlerender.fcgi?artid=147596&tool=pmcentrez&rendertype=abstract

74. Yang F, De La Fuente R, Leu NA, Baumann C, McLaughlin KJ, Wang PJ. Mouse SYCP2 is required for synaptonemal complex assembly and chromosomal synapsis during male meiosis. J Cell Biol. 2006;173:497–507. doi:10.1083/jcb.200603063

75. Yuan L, Liu J-G, Hoja M-R, Wilbertz J, Nordqvist K, Höög C. Female germ cell aneuploidy and embryo death in mice lacking the meiosis-specific protein SCP3. Science. 2002;296:1115–8. doi:10.1126/science.1070594

76. Yuan L, Liu J, Zhao J, Brundell E, Daneholt B. The Murine SCP3 Gene Is Required for Synaptonemal Complex Assembly , Chromosome Synapsis , and Male Fertility. 2000;5:73–83.

77. Dobson MJ, Pearlman RE, Karaiskakis a, Spyropoulos B, Moens PB. Synaptonemal complex proteins: occurrence, epitope mapping and chromosome disjunction. J Cell Sci. 1994;107 (Pt 1:2749–60. Available: http://www.ncbi.nlm.nih.gov/pubmed/7876343

78. Lammers JH, Offenberg HH, Aalderen M Van, Vink AC, Dietrich AJ, Heyting C. The Gene Encoding a Major Component of the Lateral Elements of Synaptonemal Complexes of the Rat Is Related to X-Linked Lymphocyte-Regulated Genes. 1994; doi:10.1128/MCB.14.2.1137.Updated

79. Pointud J-C. The intracellular localisation of TAF7L, a paralogue of transcription factor TFIID subunit TAF7, is developmentally regulated during male germ-cell differentiation. J Cell Sci. 2003;116:1847–1858. doi:10.1242/jcs.00391

80. Cheng Y, Buffone MG, Kouadio M, Goodheart M, Page DC, Gerton GL, et al. Abnormal sperm in mice lacking the Taf7l gene. Mol Cell Biol. 2007;27:2582–9. doi:10.1128/MCB.01722-06

81. Takayama T, Mishima T, Mori M, Jin H, Tsukamoto H, Takahashi K, et al. Sexually dimorphic expression of the novel germ cell antigen TEX101 during mouse gonad development. Biol Reprod. 2005;72:1315–23. doi:10.1095/biolreprod.104.038810

82. Fujihara Y, Tokuhiro K, Muro Y, Kondoh G, Araki Y, Ikawa M, et al. Expression of TEX101, regulated by ACE, is essential for the production of fertile mouse spermatozoa. Proc Natl Acad Sci U S A. 2013;110:8111–6. doi:10.1073/pnas.1222166110

83. Adelman C a, Petrini JHJ. ZIP4H (TEX11) deficiency in the mouse impairs meiotic double strand break repair and the regulation of crossing over. PLoS Genet. 2008;4:e1000042. doi:10.1371/journal.pgen.1000042

84. Yang F, Gell K, van der Heijden GW, Eckardt S, Leu NA, Page DC, et al. Meiotic failure in male mice lacking an X-linked factor. Genes Dev. 2008;22:682–91. doi:10.1101/gad.1613608

85. Hamer G, Wang H, Bolcun-filas E, Cooke HJ, Benavente R, Höög C. Progression of meiotic recombination requires structural maturation of the central element of the synaptonemal complex. 2008; doi:10.1242/jcs.033233

86. Hamer G, Gell K, Kouznetsova A, Novak I, Benavente R, Höög C. Characterization of a novel meiosis-specific protein within the central element of the synaptonemal complex. J Cell Sci. 2006;119:4025–32. doi:10.1242/jcs.03182

87. Yang F, Eckardt S, Leu NA, McLaughlin KJ, Wang PJ. Mouse TEX15 is essential for DNA double-strand break repair and chromosomal synapsis during male meiosis. J Cell Biol. 2008;180:673–9. doi:10.1083/jcb.200709057

88. Wang PJ, Page DC, McCarrey JR. Differential expression of sex-linked and autosomal germ-cell-specific genes during spermatogenesis in the mouse. Hum Mol Genet. 2005;14:2911–8. doi:10.1093/hmg/ddi322

89. Suarez PE, Rodriguez EG, Soundararajan R, Mérillat A-M, Stehle J-C, Rotman S, et al. The glucocorticoid-induced leucine zipper (gilz/Tsc22d3-2) gene locus plays a crucial role in male fertility. Mol Endocrinol. 2012;26:1000–13. doi:10.1210/me.2011-1249

90. Bruscoli S, Velardi E, Di Sante M, Bereshchenko O, Venanzi A, Coppo M, et al. Long glucocorticoid-induced leucine zipper (L-GILZ) protein interacts with ras protein pathway and contributes to spermatogenesis control. J Biol Chem. 2012;287:1242–51. doi:10.1074/jbc.M111.316372

91. Romero Y, Vuandaba M, Suarez P, Grey C, Calvel P, Conne B, et al. The Glucocorticoid-induced leucine zipper (GILZ) Is essential for spermatogonial survival and spermatogenesis. Sex Dev. 2012;6:169–77. doi:10.1159/000338415

92. Behnam B, Modarressi MH, Conti V, Taylor KE, Puliti A, Wolfe J. Expression of Tsga10 sperm tail protein in embryogenesis and neural development: from cilium to cell division. Biochem Biophys Res Commun. 2006;344:1102–10. doi:10.1016/j.bbrc.2006.03.240

93. Modarressi MH, Behnam B, Cheng M, Taylor KE, Wolfe J, van der Hoorn F a, et al. Tsga10 encodes a 65-kilodalton protein that is processed to the 27-kilodalton fibrous sheath protein. Biol Reprod. 2004;70:608–15. doi:10.1095/biolreprod.103.021170

94. Wu ATH, Sutovsky P, Xu W, van der Spoel AC, Platt FM, Oko R, et al. The postacrosomal assembly of sperm head protein, PAWP, is independent of acrosome formation and dependent on microtubular manchette transport. Dev Biol. 2007;312:471–83. doi:10.1016/j.ydbio.2007.08.051

95. Choi E, Han C, Park I, Lee B, Jin S, Choi H, et al. A novel germ cell-specific protein, SHIP1, forms a complex with chromatin remodeling activity during spermatogenesis. J Biol Chem. 2008;283:35283–94. doi:10.1074/jbc.M805590200

96. Hunt P a, Hassold TJ. Sex matters in meiosis. Science. 2002;296:2181–3. doi:10.1126/science.1071907
